# The Vulnerability Experiences Quotient (VEQ): A Study of Vulnerability, Mental Health and Life Satisfaction in Autistic Adults

**DOI:** 10.1002/aur.2162

**Published:** 2019-07-05

**Authors:** Sarah Griffiths, Carrie Allison, Rebecca Kenny, Rosemary Holt, Paula Smith, Simon Baron‐Cohen

**Affiliations:** ^1^ Autism Research Centre, Department of Psychiatry University of Cambridge Cambridge United Kingdom; ^2^ CLASS Clinic Cambridgeshire and Peterborough Mental Health NHS Foundation Trust (CPFT) Cambridge United Kingdom

**Keywords:** vulnerability, mental health, life satisfaction, depression, anxiety, adulthood, victimisation

## Abstract

Co‐morbid mental health conditions such as anxiety and depression are extremely common in autistic adults. Vulnerability to negative life experiences such as victimisation and unemployment may be partially responsible for the development of these conditions. Here we measure the frequency of negative life experiences in autistic adults and explore how these are associated with current anxiety and depression symptoms and life satisfaction. We developed the Vulnerability Experiences Quotient (VEQ) through stakeholder consultation. The VEQ includes 60 items across 10 domains. Autistic adults with a clinical diagnosis and non‐autistic controls completed the VEQ, screening measures for anxiety and depression, and a life‐satisfaction scale in an online survey. Likelihood of experiencing each VEQ event was compared between groups, using binary logistic regression. Mediation analysis was used to test whether total VEQ score mediated the relationship between autism and (1) depression (2) anxiety and (3) life satisfaction. Autistic adults (*N* = 426) reported higher rates of the majority of events in the VEQ than non‐autistic adults (N = 268). They also reported more anxiety and depression symptoms and lower life satisfaction. Group differences in anxiety, depression and life satisfaction were partially mediated by VEQ total score. This study highlights several important understudied areas of vulnerability for autistic adults, including domestic abuse, contact with social services (as parents) and financial exploitation and hardship. Improved support, advice and advocacy services are needed to reduce the vulnerability of autistic adults to negative life experiences, which may in turn improve mental health and life satisfaction in this population. ***Autism Res** 2019, 12: 1516–1528*. © 2019 The Authors. *Autism Research published by International Society for Autism Research* published by Wiley Periodicals, Inc.

**Lay Summary:**

This study investigated whether autistic adults are more vulnerable to certain negative life experiences, and whether these experiences are related to anxiety, depression and life satisfaction. We found that autistic adults are more vulnerable to many different negative life events, including employment difficulties, financial hardship and domestic abuse. Negative life experiences partially explained the higher rates of anxiety and depression symptoms and lower life satisfaction in autistic adults compared to non‐autistic adults. Improved support services are required to reduce the vulnerability of autistic adults. Reducing vulnerability may improve mental health and increase life satisfaction in this population.

## Introduction

Autism spectrum condition (hereafter ‘autism’) is a neurodevelopmental condition characterised by difficulties in social communication alongside restrictive and repetitive behaviours and interests, as well as a strong need for predictability and sensory hyper‐sensitivity [American Psychiatric Association, 2013]. There is a high rate of co‐morbid psychiatric disorders in autistic adults who do not have intellectual disability. Between 50 and 70% of these adults have a diagnosable anxiety disorder and a similar proportion have diagnosable depression [Hofvander et al., 2009; Joshi et al., 2013; Lugnegard, Hallerback, & Gillberg, 2011; Roy, Prox‐Vagedes, Ohlmeier, & Dillo, 2015].

There are a number of theories as to why individuals with autism may be more likely to experience mental health difficulties. Many of these emphasise cognitive traits, such as poor executive function [Hollocks et al., 2014], difficulties with social cognition [Eussen et al., 2013], intolerance of uncertainty [Boulter, Freeston, South, & Rodgers, 2014; Cai, Richdale, Dissanayake, & Uljarević, 2018], emotion regulation [Bruggink, Huisman, Vuijk, Kraaij, & Garnefski, 2016], and sensory sensitivities [Wigham, Rodgers, South, McConachie, & Freeston, 2015]. An alternative approach is to identify negative life events that may be a vital causal step in the path from cognitive vulnerabilities to poor mental health outcomes [Taylor & Gotham, 2016]. For example, poor executive function may only lead to anxiety if it causes an individual to be unemployed, get in to debt and become socially isolated. Concrete negative life experiences may be a more tractable and ethical target for intervention polices and practice, than altering underlying cognitive traits. It is therefore important that we have a good understanding of the types of negative life events that autistic adults are vulnerable to that may contribute to mental health difficulties.

It is well established that adverse life experiences are associated with the development of anxiety and depression in the general population [Asselmann, Wittchen, Lieb, Höfler, & Beesdo‐Baum, 2015]. Risk factors for depression and anxiety include sexual and physical abuse [Lindert et al., 2014], bullying [Arseneault, Bowes, & Shakoor, 2010]^,^ unemployment [Paul & Moser, 2009], debt [Jenkins et al., 2008] and discrimination [Almeida, Johnson, Corliss, Molnar, & Azrael, 2009]. Autistic adults are more vulnerable to many of these experiences due to social communication difficulties, which may make it harder to recognise and escape from harmful situations or relationships. In addition, appearing vulnerable or different may make them a target for exploitation, abuse and discrimination [Fisher, Moskowitz, & Hodapp, 2012]. The term ‘social vulnerability’ has been used to describe the disadvantages faced by autistic individuals and other neurodevelopmental conditions, as they try to fit into society [Jawaid et al., 2012]. Here we use the umbrella term ‘vulnerability’ to describe an increased risk of experiencing negative life events such as social isolation, unemployment, bullying and physical or sexual abuse.

Vulnerability to negative life experiences may be an important factor in the development of co‐morbid mental health conditions in autistic adults. Although studies have not looked specifically at whether vulnerability is associated with mental health symptoms in autistic adults, there is evidence that negative life events are related to depression in autistic children. Taylor and Gotham [2016] found that parent‐reported traumatic experiences were strongly related to autistic children's depression symptoms, but not anxiety symptoms and a recent longitudinal cohort study found that autistic children's experience of bullying was associated with their depression symptoms in adolescence. These studies suggest that negative experiences play a role in co‐morbid depression in autistic children.

There are no specific measures of vulnerability to negative life experiences for autistic adults. However, autistic adults who were diagnosed as children experience poorer outcomes in terms of employment, independence and social life [Hofvander et al., 2009]. Furthermore, autistic adults are more likely to have experienced bullying as children [Cappadocia, Weiss, & Pepler, 2012], sexual victimisation [Brown‐Lavoie, Viecili, & Weiss, 2014], being stopped and/or arrested by police [Rava, Shattuck, Rast, & Roux, 2017], long‐term unemployment [Howlin, 2013; Taylor, Henninger, & Mailick, 2015] and suicide attempts [Cassidy et al., 2014].

Vulnerability has been measured in autistic children using the parent‐report Social Vulnerability Questionnaire [Fisher et al., 2012], which includes questions on emotional bullying, risk awareness, social protection, perceived vulnerability, parental independence and credulity. Children with Down Syndrome, Williams Syndrome and autism all score high on this social vulnerability measure, but autistic children are particularly likely to have low social protection, leading to social isolation [Fisher, Moskowitz, & Hodapp, 2013]. This measure is limited as a measure for adults as is does not ask about adulthood vulnerability experiences such as disadvantage in employment or abuse in romantic relationships. It also contains questions which are unsuitable for conversion into a self‐report format because they would require a high degree of self‐awareness in a respondent; for example, ‘do other people perceive you as vulnerable?’.

In the current study, we used a participatory framework [Nicolaidis et al., 2011] to develop a self‐report ‘Vulnerability Experiences Quotient’ (VEQ) that measures negative life experiences that may be particularly common for autistic individuals. We aimed to select experiences that were about objective external events, for example ‘I dropped out of school/college/university’, that may be amenable to practice and policy changes.

We hypothesised that autistic adults would be more likely to experience each event in the VEQ compared to non‐autistic adults. We also hypothesised that autistic adults would report more anxiety and depression symptoms [Hofvander et al., 2009; Roy et al., 2015] and score lower on life satisfaction [Kirchner, Ruch, & Dziobek, 2016; Schmidt et al., 2015]. Additionally, we hypothesised that total score on the VEQ would be associated with more current symptoms of anxiety and depression and lower life satisfaction and that total score on the VEQ would mediate the relationship between autism diagnosis and these negative outcomes.

## Method

### *Participants and Recruitment*

Participants were autistic and non‐adults (over 18 years) who consented to take part in an online survey about autism, vulnerability and mental health. Participants were recruited *via* the Cambridge Autism Research Database (CARD) (http://www.autismresearchcentre.com) and the Cambridge Psychology participant database (http://www.cambridgepsychology.com). Participants in the CARD database are recruited because they have a diagnosis or suspected diagnosis of autism and an interest in taking part in autism research. Participants in the Cambridge Psychology Participant Database are people in the general population with an interest in taking part in psychological research. Participants were also recruited into the study through adverts placed on charity websites (e.g. http://www.autistica.org.uk) and on social media (e.g. Twitter).

### *Measures*

***Demographic information***. Demographic information was collected at the start of the online survey (see Supporting Information). Information on diagnoses was collected by asking participants to select from a list of conditions they had received from a clinician. Participants were then asked to select conditions they suspected they had but with which they had not been formally diagnosed.

*Life satisfaction* was assessed using the Satisfaction with Life Scale (SWLS; Diener, Emmons, Larsen, & Griffin, 1985). Participants indicate their agreement with five statements, such as ‘so far I have got the important things I want in life’, on a 7‐point Likert scale (1 = ‘strongly disagree’ to 7 = ‘strongly agree’). Responses are averaged to give a total score, with higher scores indicating greater life satisfaction. This scale has previously been used in populations of autistic adults [Kirchner et al., 2016; Mazurek, 2013] and has shown very good internal reliability (Cronbach's alpha = 0.80–0.89).

*Depression and anxiety symptoms* were assessed using the Patient Health Questionnaire subscales for anxiety (GAD7; Spitzer, Kroenke, Williams, & Lowe, 2006) and depression (PHQ9; Kroenke, Spitzer, & Williams, 2001). These have been widely used and validated as screening measures of depression and anxiety symptoms. The anxiety scale includes seven items and the depression scale includes nine items. Participants report how often they have experienced symptoms in the previous 2 weeks on a 4‐point Likert scale (1 = not at all to 4 = nearly every day). These scales have previously been used in autistic adults and have shown very good internal validity (Cronbach's α = 0.86 for PHQ9 and α = 0.88 for GAD7; Mazurek, 2013).

*Autistic traits* were measured using the short version of the Autism Spectrum Quotient (AQ‐10) [Allison, Auyeung, & Baron‐Cohen, 2012]. This version includes 10 items from the original AQ [Baron‐Cohen, Wheelwright, Skinner, Martin, & Clubley, 2001] which was developed as a quantitative measure for autistic traits in the general population. Participants are asked how much they agree with 10 statements about themselves (e.g. I know how to tell if someone listening to me is getting bored). Participants say whether they ‘definitely agree’, ‘slightly agree’, ‘slightly disagree’ or ‘definitely disagree’. A score of 0 is given for ‘definitely agree’ and ‘slightly agree’ and a score of 1 is given for ‘slightly disagree’ and ‘definitely disagree’. Four of the items are reverse scored. A score on the AQ‐10 of six or above may indicate an individual warrants a clinical diagnostic assessment. At that cut‐off point, the AQ‐10 has good sensitivity (0.88) and specificity (0.91) for detecting autism in a diagnosed sample. The AQ‐10 was recently used in a very large online study of 600,000 non‐autistic and 36,000 autistic adults, showing robust group differences [Greenberg, Warrier, Allison, & Baron‐Cohen, 2018], replicating earlier big data studies of the full AQ [Ruzich et al., 2015].

The *Vulnerability Experiences Quotient (VEQ)* was developed using a consultation process that included researchers, autistic adults and clinicians with experience working with autistic adults in the UK National Health Service. The research team started by reviewing the literature on life experiences that are risk factors for mental health conditions and then ran meetings with an advisory board of autistic adults (*N* = 8). The advisory board helped determine which negative life events were particularly relevant to autistic people, and gave feedback on the wording of the survey items. Feedback on the survey items was also provided *via* email by clinicians. This participatory approach was used to ensure that the measure was acceptable for autistic individuals and aligned with community priorities for research in this area [Nicolaidis et al., 2011]. This approach leads to the inclusion of some items that have not previously been studied in the autism literature, including questions on contact with social services, domestic abuse and self‐medication.

The final survey included 60 items relating to 10 potential areas of vulnerability: *1. education, 2. employment, 3. finances, 4. interactions with social services, 5. interactions with the criminal justice system, 6. childhood victimisation, 7. adulthood victimisation, 8. domestic abuse, 9. mental health* and *10. social support*. Each area had between three and nine items. Items were presented in a random order. Each item was a statement about a life experience, e.g. ‘There was a period in my life where I had nowhere safe to live’. Most items were statements of negative experiences, but the three social support items were positive experiences to avoid a relentlessly negative focus and to explore potentially protective factors. For example: ‘There has always been someone in my life who would try to help me if I was in trouble’. Participants were asked to report whether they had had each experience by selecting ‘yes’, ‘no’ or ‘no opportunity’. At the end of the checklist there was an open text box for participants to add details of any other negative experiences they felt were relevant. Participants scored 1 for ‘yes’, and 0 for ‘no’ and ‘no opportunity’ for all items, except the three social support items which were reverse scored (0 for ‘yes’ and 1 for ‘no’ or ‘no opportunity’). Total score was between 0 and 60. The 60‐item VEQ was found to have very good internal validity in both the autistic (Cronbach's α = 0.89) and control (Cronbach's α = 0.88) group in the current study.

This study was approved by the Psychology Research Ethics Committee, University of Cambridge (PRE.2017.031).

### *Design and Analysis*

This was a cross‐sectional study comparing autistic individuals to a non‐autistic control group. Participants in the autism group had a diagnosis of autism from a recognised qualified clinician (psychiatrist, clinical psychologist, neurologist, paediatrician). Participants in the control group neither reported an autism diagnosis, nor suspected they had autism. Participants who reported that they suspected they were autistic but did not have a clinical diagnosis, were excluded from both groups. Dependent variables were responses on each item of the VEQ, total VEQ score and total scores on the SWLS, GAD7 and PHQ9 scales. We also used scores on the AQ‐10 as a continuous measure of autistic traits to assess whether autistic traits were associated with VEQ score within each group.

Participants who indicated that they did not have children were excluded from the analysis of individual items in the ‘social service contact’ domain, participants who indicated that they had never worked were excluded from analysis of individual items in the ‘work’ domain (except for the one item about seeking work), and participants who indicated that they had never been in a relationship were excluded from individual analysis of items in the ‘domestic abuse’ domain. For analysis of the VEQ total score, all participants were included. Participants who responded to less than 95% of the VEQ items were excluded from all analysis. Participants were excluded from analysis of individual items in the VEQ if their responses were missing. Missing responses were replaced with 0 in calculation of the total VEQ score.

All statistical analyses were performed using IBM SPSS version 25. T‐tests were used to compare group mean scores on the GAD7, PHQ9, SQW and VEQ. T statistics and *P*‐values were adjusted for unequal variances where necessary. Binary logistical regression was used to compare the likelihood of each of experience in the VEQ between the two groups. *P*‐values were adjusted for 60 comparisons using the Bonferroni correction.

The mediation effect of VEQ on relationships between (1) autism diagnosis (coded as autism diagnosis = 1, no diagnosis = 0) and anxiety, (2) autism diagnosis and depression, and (3) autism diagnosis and life satisfaction were calculated using the PROCESS macro [Hayes, 2012]. First, a simple linear regression was performed with diagnosis as the predictor (dummy coded as controls = 1 and autism = 2) and total VEQ score as the outcome. Second, three separate linear regression models were calculated with autism as the predictor and SWL, GAD7 and PHQ9 as outcomes. Third, VEQ was entered as a mediator into these three models using PROCESS Model 4, with 5000 bootstrap samples drawn to estimate direct and indirect effects of autism diagnosis on the outcome variables [Preacher & Hayes, 2008]. Bootstrapping provided 95% confidence intervals (CI) around the indirect effects. If confidence intervals do not cross zero, this indicates that VEQ is a significant mediator in the relationship between autism and the outcome variable.

## Results

### *Participants*

Eight hundred and eighty‐six participants consented to take part in the study; 83 of these participants did not complete any items in the survey after consenting to take part. Of the remaining 803 participants, 446 (56%) participants reported a clinical diagnosis of autism, and 288 (35%) reported no diagnosis or suspected diagnosis of autism. Sixty‐nine (9%) participants (41 female, 22 male, 6 other) reported that they suspected they were autistic, so were excluded from the analysis. An additional 40 participants were excluded because they completed <95% of the VEQ, leaving 426 participants in the autistic group, and 268 in the control group. Excluded participants did not differ significantly from the final sample in autism diagnostic category (*X* ^2^(1) = 2.06, *P* = 0.15), gender (*X* ^2^(2) > 0.01, *P* > 0.99), age (*t*(729) = 0.98, *P* = 0.33) or highest qualification (*X* ^2^(4) = 8.14, *P* = 0.09). Of the final sample 66% were living in the United Kingdom, 18% in the USA, 6% in Australia, New Zealand or Canada, 7% in other European countries and 2% elsewhere.

Demographic information about the groups is summarised in Table 1. Participants were classified as ‘male’ or ‘female’, if they reported that their gender assigned at birth (male/female) matched their current gender identity, or ‘other/transsexual/non‐binary’, if their assigned gender was different to their gender identity (male/female/non‐binary/other). There were more females than males in both groups but the gender imbalance was greater in the control group than the autistic group. There was a wide age range in both groups but the average age of the control group was older than the autistic group. All analyses were conducted both with and without adjustment for age and gender. In adjusted analyses, individuals who did not report their age (*n* = 3) and those whose gender was transgender/non‐binary/other (*n* = 53) were excluded from the analysis. Where results do not differ qualitatively we report only the unadjusted analyses to maximise statistical power.

**Table 1:** Demographic Information for the Autism and Control Groups

	Autism (*N* = 426)	%	Control (*N* = 268)	%
Age				
Mean (SD)	44 (14.37)		51 (15.33)	
AQ‐10				
Mean (SD)	7.79 (2.03)		2.83 (2.35)	
Above cut off	361	85	35	13
Sex/Gender				
Male	174	40	71	26
Female	202	47	194	72
Transgender/non‐binary/other	50	12	3	1
Employment status				
Fulltime paid	104	24	81	30
Part‐time paid	68	16	44	17
Voluntary	39	9	14	5
Student	68	16	24	9
Retired	44	10	64	24
Seeking work	41	10	8	3
Unable to work	96	23	18	7
Self‐employed	52	12	42	16
Carer/homemaker	31	7	22	8
Ever held paid employment	386	90	260	97
Highest qualification				
Postgraduate level	144	34	105	39
Undergraduate level	119	28	76	28
Vocational qualification	82	19	46	17
School level	72	17	38	14
No formal qualification	9	2	3	1
Attended SEN school	29	7	8	3
Extra help at school	78	18	18	7
Relationship status				
Single	194	46	61	23
Married/Civil partnership	127	30	142	53
Cohabiting	82	19	45	17
Long‐term relationship (not cohabiting)	40	9	19	7
Divorced/Separated	48	11	30	11
Widowed	0	0	4	1
Ever been in a relationship	354	83	257	95
Children	147	35	170	63
Living situation				
With parents	83	19	11	4%
With partner	156	37	159	59
With other family members	24	6	11	4
With children	82	19	89	33
Shared accommodation	17	4	7	3
With friends	15	4	10	4
Alone	136	32	52	19
Support with household activities	196	46	74	28

### *Psychiatric Diagnoses*

Table 2 lists diagnoses reported by each group. The most common diagnosis in both groups was depression, followed by anxiety disorder. Both of these diagnoses were around three times more common in the autistic group compared to the control group. Only 3% of the autism group had been given a diagnosis of intellectual disability which is very low compared to the autism population as a whole which is estimated to be 55% [Charman et al., 2011], but expected in an online survey study.

**Table 2:** Diagnoses Given by a Clinician for the Autism and Control Groups

	Autism (*N* = 426)		Control (*N* = 268)	
	*N*	%	*N*	%
Alcohol abuse	23	5	8	2
Anxiety disorder	198	43	59	13
ADHD	63	13	10	2
Bipolar disorder	20	4	6	1
Conduct disorder	2	<1	0	0
Depression	294	64	106	23
Dyslexia	37	8	10	2
Dyspraxia	39	9	3	1
Eating disorder	30	7	16	4
Intellectual disability	15	3	1	0
General anxiety disorder	96	21	28	6
Language delay	19	4	4	1
Obsessive compulsive disorder	53	11	8	2
Oppositional defiance disorder	3	<1	0	0
Panic disorder	30	7	10	2
Personality disorder	46	10	3	1
Post‐traumatic stress disorder	73	16	16	4
Schizophrenia/Psychosis	19	4	6	1
Sensory processing disorder	47	10	4	1
Social phobia	69	15	10	2
Specific phobia	14	3	1	<1
Tourette syndrome	7	2	1	<1
None (apart from autism)	58	14	129	48

### *Group Differences in Current Mental Health Symptoms, Life Satisfaction and Vulnerability Experiences*

Table 3 shows the mean scores on the SWL, GAD7, PHQ9 and VEQ for each group. The autistic group report reduced levels of life satisfaction and elevated levels of anxiety and depression symptoms and a greater number of vulnerability experiences in comparison to the control group. Cohen's *d* statistics suggest that all of these effects are large.

**Table 3:** Scores on SWL, GAD7, PHQ9 and Total VEQ by Group

	Autism	Control					
	M (SD)	M (SD)	Difference	df	*t*	*P*	*d*
SWL	16.18 (7·55)	22.77 (7·60)	6.57	692	11.16	<0.001	0.87
GAD7	9.34 (6.07)	4.75 (4.99)	5.40	645	11.01	<0.001	0.86
PHQ9	11.39 (7.07)	5.99 (5.75)	4.77	648	11.20	<0.001	0.84
VEQ	22.54 (10.37)	11.06 (8.49)	11.48	646	15.91	<0.001	1.21

*Note*. Independent sample *t*‐tests are reported for the group comparison. Welshes *t*‐statistics and adjusted *P* values are reported for GAD7, PHQ9 and VEQ as the groups had unequal variance.

Table 4 shows the number of people in each group who reported that they had each type of vulnerability experience in the VEQ. Unadjusted regression models provided evidence for group differences for 51 out of the 60 events after Bonferroni correction for multiple comparisons (in bold in Table 4).

**Table 4:** Percentages of Participants Who Responded ‘Yes’ for Each Item on the VEQ for Each Group

	Item	Autism	Control	χ^2^ (Wald)		Odds ratio (95% CI)
Education	**I dropped out of school/college/university**	38% (162/426)	19% (52/268)	25.89	*P* < 0.001	2.55 (1.78–3.66)
	**I missed more than 2 weeks of school/college/university due to anxiety or depression**	47% (199/426)	15% (39/268)	68.07	*P* < 0.001	5.15 (3.49–7.60)
	**I was temporarily or permanently excluded from school/college/university**	14% (61/426)	3% (8/268)	19.35	*P* < 0.001	5.43 (2.56–11.54)
	**My parents/carers tried to get additional support for me at school but the school did not provide any**	19% (80/426)	7% (19/268)	17.06	*P* = 0.002	3.03 (1.79–5.13)
	I left a school/college/university without a qualification because I failed my exams	15% (65/426)	8% (22/268)	7.24	*P* = 0.429	2.01 (1.21–3.35)
	**I avoided attending lessons or lectures at school/college/university because I found them stressful**	54% (229/425)	17% (45/268)	85.25	*P* < 0.001	5.79 (3.99–8.41)
Employment	**I was signed off from work for at least 2 months due to anxiety, depression or any other mental health reason**	43% (164/386)	15% (38/260)	51.62	*P* < 0.001	4.32 (2.90–6.43)
	**I spent at least a year unemployed and seeking work**	48% (189/426)	15% (40/268)	66.73	*P* < 0.001	5.12 (3.46–7.57)
	**I was sacked from a job**	42% (162/386)	24% (61/260)	22.99	*P* < 0.001	2.36 (1.66–3.35)
	**Disciplinary action was taken against me at work**	30% (115/386)	14% (35/260)	22.18	*P* < 0.001	2.73 (1.80–4.14)
	**I left a job because I was unable to deal with the work environment and/or the demands of the job**	73% (280/386)	32% (84/260)	95.70	*P* < 0.001	5.54 (3.93–7.80)
	**I have been regularly overlooked for promotions or contract renewals at work**	33% (128/386)	12% (30/260)	36.15	*P* < 0.001	3.80 (2.46–5.88)
	**I left a job because I was being treated badly by colleagues**	49% (188/386)	19% (49/260)	55.83	*P* < 0.001	4.09 (2.83–5.92)
	**I have been unable to get a job which matches my level of training and qualification**	55% (211/386)	21% (54/259)	68.35	*P* < 0.001	4.58 (3.19–6.57)
Finances	I have had possessions forcibly removed by debt collectors	4% (16/426)	4% (10/268)	0.00	*P* = 1.00	1.01 (0.45–2.25)
	**There has been a period in my life where I did not have enough money to meet my basic needs (e.g.· food, rent, medical care)**	45% (193/426)	25% (67/268)	28.21	*P* < 0.001	2.49 (1.78–3.48)
	**There has been a period in my life where I had debts (other than a mortgage or student loan) that were greater than my yearly income**	26% (109/426)	13% (35/268)	15.18	*P* = 0.006	2.29 (1.51–3.47)
	**I had to leave my home because I was unable to keep up with mortgage or rent payments** †	12% (52/426)	4% (11/268)	11.89	*P* = 0.034	3.25 (1.66–6.35)
	**There was a period in my life where I had nowhere safe to live**	27% (114/426)	11% (30/268)	22.88	*P* < 0.001	2.90 (1.87–4.48)
Social services	My child/ren were subject to a child protection investigation due to concerns about my ability to care for them	9% (13/147)	2% (3/170)	6.71	*P* = 0.574	5.40 (1.51–19.34)
	My child/ren were referred to social services due to concerns about my ability to care for them	14% (21/147)	<1% (1/170)	10.50	*P* = 0.072	28.17 (3.74–212.18)
	I lost custody of my child/ren through court proceedings due to concerns about my ability to care for them	4% (6/147)	<1% (1/170)	3.30	*P* = 1.00	7.19 (0.86–60.44)
	**An educational, medical or social work professional questioned my ability to care for my child**	19% (28/147)	4% (7/170)	14.98	*P* = 0.007	5.48 (2.32–12.96)
Criminal justice system	I have a criminal record	10% (41/426)	4% (10/268)	7.81	*P* = 0.312	2.75 (1.35–5.58)
	I was charged with a criminal offense (not including speeding or parking fines)	14% (60/426)	8% (22/268)	5.33	*P* = 1.00	1.83 (1.10–3.07)
	**I was cautioned by the police (not including cautions for minor traffic offences)**	18% (77/426)	6% (15/268)	19.97	*P* < 0.001	3.72 (2.09–6.62)
	I spent time in prison or a juvenile detention centre	3% (12/426)	2% (5/268)	0.43	*P* = 1.00	1.53 (0.53–4.38)
	**I was arrested by the police** †	18% (77/425)	9% (23/268)	11.59	*P* = 0.040	2.36 (1.44–3.86)
Childhood victimisation	**As a child, other children bullied me**	87% (370/426)	53% (143/268)	86.50	*P* < 0.001	5.78 (3.99–8.36)
	**As a child, an adult hurt me badly enough that it left marks on my body**	29% (125/426)	16% (44/268)	14.55	*P* = 0.008	2.11 (1.44–3.11)
	**As a child, other children left me out of activities**	85% (360/426)	46% (123/268)	105.09	*P* < 0.001	6.43 (4.51–9.18)
	**As a child, children spread rumours about me or talked about me behind my back**	77% (328/425)	45% (119/267)	72.35	*P* < 0.001	4.21 (3.02–5.86)
	**As a child, another child hurt me badly enough that it left marks on my body (e.g. bruises or scratches)**	51% (215/426)	25% (66/268)	43.86	*P* < 0.001	3.12 (2.23–4.37)
	**As a child, children called me names or insulted me**	85% (362/425)	57% (151/267)	65.08	*P* < 0.001	4.41 (3.08–6.33)
	**As a child, an adult humiliated, embarrassed or scared me**	79% (334/425)	52% (139/268)	51.93	*P* < 0.001	3.41 (2.44–4.75)
	As a child, an adult touched me in a sexual way, or tried to make me touch them in a sexual way†	30% (128/425)	20% (54/268)	8.34	*P* = 0.233	1.71 (1.19–2.46)
	**As a child, an adult swore at me or called me names like stupid, ugly or lazy**	63% (268/426)	33% (89/268)	56.02	*P* < 0.001	3.41 (2.47–4.70)
Adulthood victimisation	**I have been bullied by someone in my family**	55% (234/426)	34% (90/267)	29.12	*P* < 0.001	2.40 (1.75–3.29)
	**I have been pressured into sexual activity**	44% (185/425)	23% (60/267)	30.79	*P* < 0.001	2.66 (1.88–3.76)
	**I have been bullied by someone at work**	54% (231/426)	36% (97/268)	21.17	*P* < 0.001	2.09 (1.53–2.86)
	**I have been tricked or pressured into breaking the law**	21% (91/426)	6% (17/268)	25.13	*P* < 0.001	4.01 (2.33–6.90)
	**I have been physically forced into sexual activity**	26% (111/425)	15% (39/268)	12.64	*P* = 0.023	2.08 (1.39–3.11)
	**As an adult, I have been hurt by someone badly enough that it left marks on my body (e.g. bruises or scratches)**	34% (145/426)	18% (48/268)	20.68	*P* < 0.001	2.37 (1.63–3.43)
	**I have been bullied by someone that I considered to be a friend**	70% (299/426)	31% (84/268)	94.22	*P* < 0.001	5.16 (3.70–7.18)
	**I have been tricked or pressured in to giving someone money or possessions**	48% (205/426)	20% (54/267)	51.59	*P* < 0.001	3.66 (2.57–5.21)
Domestic abuse	**My partner forced me into sexual activity**	20% (72/354)	9% (22/257)	14.99	*P* = 0.006	2.73 (1.64–4.53)
	**My partner physically hurt me (e.g.· shoved, slapped or punched me)**	30% (105/354)	18% (45/257)	11.64	*P* = 0.039	1.99 (1.34–2.95)
	**My partner threatened to harm me or to harm someone I care about**	22% (78/354)	11% (27/257)	13.35	*P* = 0.016	2.41 (1.50–3.86)
	**My partner took advantage of me financially**	25% (88/354)	11% (29/257)	16.92	*P* = 0.002	2.60 (1.65–4.10)
	**My partner humiliated or embarrassed me**	39% (138/354)	23% (59/255)	16.69	*P* = 0.003	2.12 (1.48–3.05)
Mental illness	**There was a period of my life where I was regularly using alcohol or another (non‐prescribed) drug in order to cope·**	39% (166/426)	25% (66/268)	14.97	*P* = 0.007	1.95 (1.39–2.74)
	**I was incorrectly diagnosed with a mental health condition (e.g. ADHD instead of autism)**	40% (171/426)	5% (13/268)	73.27	*P* < 0.001	13.15 (7.29–23.73)
	**I have been sectioned because of a mental health condition** †	10% (43/426)	2% (6/268)	12.87	*P* = 0.020	4.90 (2.06–11.68)
	**I have had a mental health condition that affected my daily life**	84% (356/426)	38% (103/268)	133.89	*P* < 0.001	8.15 (5.71–11.62)
	**I have made suicide plans**	60% (255/426)	26% (70/267)	70.62	*P* < 0.001	4.20 (3.00–5.86)
	**I have attempted suicide**	41% (174/426)	13% (34/268)	55.97	*P* < 0.001	4.75 (3.16–7.15)
	**I have deliberately harmed myself**	60% (259/426)	20% (54/266)	98.63	*P* < 0.001	6.09 (4.26–8.70)
Social support	**There has always been someone in my life who would try to help me if I was in trouble**	56% (238/426)	76% (203/268)	27.33	*P* < 0.001	0.41 (0.29–.057)
	**There has always been someone in my life who would care for me if I was ill**	52% (220/426)	73% (196/268)	30.84	*P* < 0.001	0.39 (0.28–0.55)
	**I have always known that there is someone in my life who loves me**	54% (228/426)	76% (203/267)	34.24	*P* < 0.001	0.36 (0.26–0.51)

† Indicates a change in statistical significance in the analysis adjusted for age and sex (see Supporting Information for results of adjusted analysis).

*Note*. Wald statistics and odds ratios for the group difference. *P* values have been adjusted for the 60 multiple comparisons using the Bonferroni correction. Items for which there was a significant group difference are in bold font.

When regression models were adjusted for age and sex, there was evidence for group differences on 49 out of 60 items. Group differences for three items became non‐significant after adjustment; ‘I had to leave my home because I was unable to keep up with mortgage or rent payments’, (*P* = 0.48), ‘I was arrested by the police’ (*P* = 0.34) and ‘I have been sectioned because of a mental health condition’ (*P* = 0.14). Conversely, one item became significant after adjustment ‘As a child, an adult touched me in a sexual way, or tried to make me touch them in a sexual way’ (*P* = 0.027). Please see Supporting Information for the complete statistics for the adjusted models.

### *Correlations between Autism, Mental Health Symptoms, Life Satisfaction and Vulnerability to Negative Experiences*

Autism, mental health symptoms, life satisfaction and vulnerability to negative experiences are significantly correlated with medium to large effect sizes (see Table 5). We used the binary categorical variable of autism diagnostic group in our analysis, however, we also looked at whether autistic traits (AQ‐10 scores) were associated with vulnerability experiences (VEQ) within the autism and control groups. We found moderate correlations in each (autistic, *r* = 0.30, *P* < 0.001, control *r* = 0.40, *P* < 0.001), suggesting that both autistic and non‐autistic individuals with fewer autistic traits experience fewer negative life events than those with more autistic traits.

**Table 5:** Pearson Correlation Coefficients for Associations between Predictor and Outcome Variables

	VEQ	SWL	GAD7	PHQ9
Autism	·50*	−.39*	.38*	.37*
VEQ	—	−.53*	.52*	.55*
SWL		—	−.53*	−.64*
Anxiety			—	.75*

*p < 0.001.

### *Vulnerability Experiences as a Mediator of the Relationship between Autism, Current Mental Health Symptoms and Life Satisfaction*

**Figure 1:**
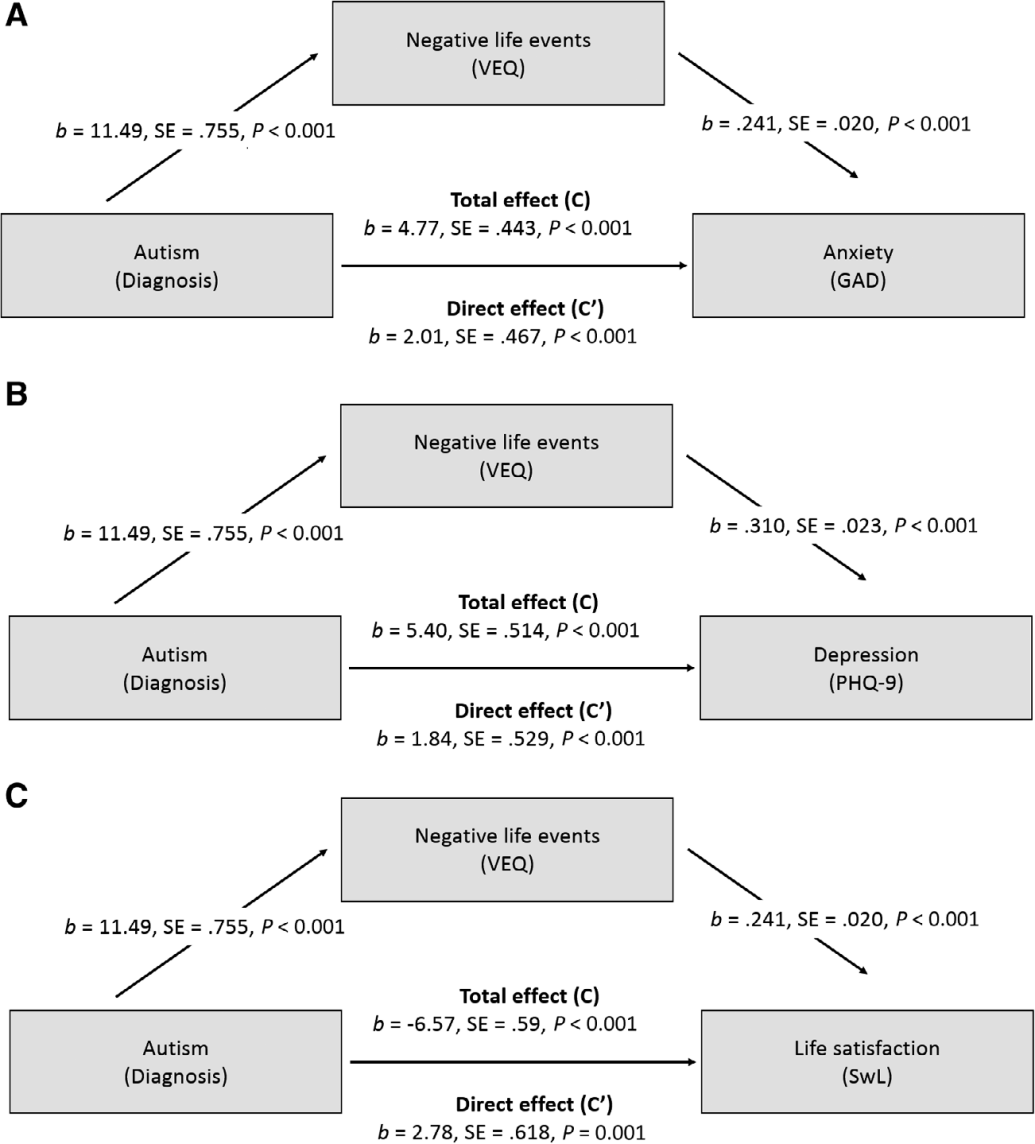
Mediation pathways for (A) anxiety symptoms, (B) depression symptoms and (C) satisfaction with life.

Linear regression confirmed that autism diagnosis was associated with score on the VEQ (F[1,692] = 231·25, *P* < ·001, *R* ^2^ = ·250). Separate simple linear regression models showed that autism diagnosis was associated with life satisfaction, (F[1,692] = 124·47, *P* < ·001, *R* ^2^ = ·152), anxiety symptoms (F[1,692] = 116·21, *P* < ·001, *R* ^2^ = ·144) and depression (F[1,692] = 110·51, *P* < ·001, *R* ^2^ = ·138). When VEQ score was added as a mediator in these models, the total amount of variance explained increased from 15% to 31% for life satisfaction (F(1,692) = 151·69, *P* < ·001, *R* ^2^ = ·305), from 14% to 29% for anxiety (F[1,692] =139·80, *P* < ·001, *R* ^2^ = ·288) and from 14% to 32% for depression (F[1,692] = 160·16, p < ·001, R^2^ = ·317). Boot strapping estimates indicated that VEQ was a partial mediator in the relationships between autism and life satisfaction (indirect effect estimate *b* = −3·81, 95% CI = −4·48, −3·14), autism and anxiety (indirect effect estimate *b* = 2·76, 95% CI = 2·22, 3·32) and autism and depression (indirect effect estimate *b* = 3·56, 95% CI = 2·95, 4·23). Beta coefficients and standard errors for the regression equations are shown in Figure 1. Note that the effect sizes of the ‘total effects’ of diagnosis on the outcomes variables are much smaller than the effect sizes for the ‘direct effects’ (after controlling for the mediator), giving evidence for partial mediation. Adjusting for age and sex in these models did not qualitatively change these results.

## Discussion

This cross‐sectional study measured vulnerability of autistic adults to a variety of negative life experiences using a newly developed VEQ. The VEQ was designed based on the literature on risk factors for mental health conditions, and using a participatory research approach [Nicolaidis et al., 2011], to measure negative experiences that autistic adults felt impacted on their mental health. Autistic adults were more likely than non‐autistic adults to have experienced the majority of the events assessed by the VEQ, demonstrating their significant vulnerability in society. Furthermore, autistic traits, measured using the AQ‐10, were associated with experiencing a greater number of negative life experiences in the VEQ in individuals with and without an autism diagnosis.

We found an association between vulnerability experiences and current anxiety symptoms, depression symptoms and life satisfaction in autistic and non‐autistic adults. As expected, autistic adults had higher rates of depression and anxiety symptoms [Joshi et al., 2013; Mazurek, 2013; Roy et al., 2015], and lower life satisfaction [Kirchner et al., 2016; Schmidt et al., 2015] than non‐autistic adults. A mediation analysis suggests that these group differences may be partially due to greater vulnerability to negative life experiences in the autistic group. Although we cannot determine the direction of causality from this study, future longitudinal studies should test whether victimisation and other negative life experiences are a cause of high rates of co‐morbid anxiety and mood disorders and lower life satisfaction in autistic adults.

Our findings highlight several important understudied areas of vulnerability for autistic adults. First, as well as confirming previous findings that autistic children are often bullied by peers [Cappadocia et al., 2012], our study also found high rates of other types of victimisation. An alarmingly high number of autistic adults reported having been victimised physically, verbally, emotionally and sexually by adults when they were children. This is in line with a recent study that found that autism diagnosis was associated with parent‐reported experience of maltreatment [Dinkler et al., 2017]. We also found that autistic adults who had been in a relationship were more likely to have been sexually, physically, financially and emotionally abused or threatened by a partner compared to non‐autistic adults in relationships. We believe this is the first study to report an association between autism and domestic abuse. The lack of previous research in this area may be due to the belief that few autistic people have romantic relationships. However, in our sample of intellectually able autistic adults, 83% had been in a romantic relationship, suggesting that many autistic adults are potentially vulnerable to domestic abuse.

A second understudied area of vulnerability explored in this study is financial hardship and exploitation. High numbers of autistic adults reported financial difficulties, including having nowhere safe to life. These difficulties may result from financial exploitation, as well as unemployment, given almost half of our sample reported being tricked or pressured in to giving someone money or possessions. Studies have shown that parents of autistic children experience financial difficulties [Sharpe & Baker, 2007] and vulnerability to financial victimisation has been reported in adults with intellectual disability [Gillian, Lynn, Kenneth, Michael, & Priscilla, 2017] but this is the first study to show the extent of financial difficulties for autistic adults. Our finding that many autistic adults have housing difficulties is in accordance with a recent study that found high levels of autistic traits in a homeless population [Churchard, Ryder, Greenhill, & Mandy, 2019].

A third unexplored area of vulnerability investigated in this study was parent contact with social services; a topic suggested by our advisory group. Nineteen per cent of autistic parents, around four times as many as in the non‐autistic group, reported that their ability to care for their child had been questioned by a professional. There was no statistical evidence that autistic parents were more likely to experience referral to social services, a child protection investigation, or have their child removed by social services than non‐autistic adults. This may be due to insufficient power to detect group differences for these rarer events. Nonetheless, it is important that further research looks at why the parenting of autistic adults is being questioned and how autistic parents can be supported without feeling judged.

As well as highlighting some relatively understudied areas of vulnerability, our study also found high incidence of some well‐established vulnerabilities. In line with previous studies [Taylor et al., 2015], we found evidence of substantial difficulties in employment. Although 90% of our sample had held paid employment, rates of negative experiences such as long‐term unemployment and losing jobs were high. Similarly, although 62% had university level qualification, many reported experiences of difficulty within education, for example missing lessons due to anxiety and depression or stress. This demonstrates that individuals that might be considered ‘high‐functioning’ are vulnerable to negative events in education and employment that may affect their mental health.

Similarly in line with previous research [Rava et al., 2017], we found that autistic adults are at high risk of being cautioned and possibly arrested by police. However, we did not find that autistic adults were more likely to have been charged with a criminal offence, to hold a criminal record, or to have spent time in prison than non‐autistic adults. Again, this may be due to insufficient statistical power to detect differences between groups for these rarer events. Alternatively, it may suggest that autistic individuals are more likely to attract police attention, perhaps due to unusual behaviours, but are not more likely to commit crimes. Either way, this finding highlights the importance of autism awareness training for police [Crane, Maras, Hawken, Mulcahy, & Memon, 2016].

Negative experiences related to mental health were very common in our autistic group. Perhaps most strikingly, 60% reported having made suicide plans, 41% reported making a suicide attempt and 64% reported self‐harming. This is higher than a previous estimate from a study of recently diagnosed adults that reported 35% had experienced suicidal plans or attempts [Cassidy et al., 2014]. This may be explained by the higher prevalence of depression (63%) in our sample compared to the previous study (32%) [Cassidy et al., 2014]. There was also evidence of experiencing difficulties with getting diagnoses of co‐morbid conditions, with 40% of autistic adults compared to 5% of non‐autistic adults reporting having been misdiagnosed with a mental health condition.

In this article we do not explore the reasons that autistic adults are more likely to experience each event measured by the VEQ. Given the broad range of events covered it is likely that there are many different predictors and that these differ for each event. For example, the items in the education section will depend on support available at the school or college that the individual attended. Future research should look to determine the specific risk factors for each event to identify ways of reducing risk of these events through policy and practice changes. Individual difference in cognitive traits such as IQ, social cognition, tolerance of uncertainty, emotion regulation, and sensory sensitivities may also contribute to an individual's vulnerability to each event. These cognitive factors have been linked to negative mental health outcomes [Boulter et al., 2014; Bruggink et al., 2016; Cai et al., 2018; Eussen et al., 2013; Hollocks et al., 2014; Wigham et al., 2015], but their influence may be partially mediated by increasing vulnerability to environmental triggers. Interventions aimed at reducing negative events identified in this study may therefore work to mitigate the impact of these cognitive factors on mental health.

## Limitations

There are limitations to this study that should be considered. First, our groups were not matched for age and gender. The autistic group included more males and was slightly older than the control group. After controlling for age and gender in our analyses, some of the group differences for specific events were no longer statistically significant. This suggests that these events are more common for autistic individuals partially because they are more common in men (e.g. being arrested). Conversely, there was one item on sexual abuse in childhood for which evidence for a group difference increased after controlling for gender, possibly because this experience is more common for women. However, even after controlling for gender and age, the overall picture of high rates of negative life experiences remained, as did the association between number of negative experiences and mental health outcomes, suggesting that while vulnerability may manifest slightly differently for men and women, autism increases vulnerability to negative life events in both genders. Second, the control group may not have been representative of the general population, as they reported high rates of diagnoses of mental health conditions and higher than average scores on the AQ. However, if we did have a more representative control group, the group differences in mental health symptoms and life experiences would likely be even larger. Third, the VEQ contains a number of items that are only applicable to some individuals; for example, the domestic abuse items are not relevant for people who have never been in a relationship. This means that the total score on the VEQ may underestimate the potential vulnerability of the autistic individuals who have less opportunity to experience some of the events. Finally, this is a cross‐sectional study so it is not possible to determine direction of causality. Although our findings are consistent with the hypothesis that vulnerability to negative life events contributes to higher rates of anxiety, depression and lower life satisfaction in autistic adults, it is almost certainly a bidirectional relationship in which these mental health conditions also cause vulnerability to negative experiences (e.g. make one more likely to be sectioned or lose a job). Further longitudinal studies would be needed to determine the nature of causal relationships.

## Implications

The findings from this study are relevant for service providers and policy makers as they highlight areas where resources should be focused. Some service providers in the United Kingdom offer ‘low‐level’ support to autistic adults that includes practical assistance with daily life, vocational support, training (e.g. financial management, safely awareness) and facilitating access to services [Lorenc et al., 2016]. Future research should systematically evaluate whether support services are effective in reducing vulnerability of autistic adults. Beyond education and practical assistance, peer mentoring or support groups may be effective in reducing vulnerability by increasing social support [Lauren, Carla, & Shaun, 2017]. Our findings indicate that few autistic adults have good social support networks; only half of autistic adults reported that there was always someone who would help them if they were in trouble, suggesting that this type of intervention could be beneficial.

## Supporting information


**Appendix S1**: Supporting Information.

**Table S1**: Logistical Regression Analyses for Each Item in the VEQ Adjusted for Age and Sex. Excludes participants who did not report their age (*n* = 3) and those whose gender was transgender/non‐binary/other (*n* = 53). *P* values have been adjusted for the 60 multiple comparisons using the Bonferroni correction. Items for which there was a significant group difference are in **bold font**.

## References

[aur2162-bib-0001] Allison, C. , Auyeung, B. , & Baron‐Cohen, S. (2012). Toward brief ‘Red Flags’ for autism screening: The Short Autism Spectrum Quotient and the Short Quantitative Checklist for Autism in toddlers in 1,000 cases and 3,000 controls [corrected]. Journal of the American Academy of Child and Adolescent Psychiatry, 51(2), 202–212.e207. 10.1016/j.jaac.2011.11.003 22265366

[aur2162-bib-0002] Almeida, J. , Johnson, R. M. , Corliss, H. L. , Molnar, B. E. , & Azrael, D. (2009). Emotional distress among LGBT youth: The influence of perceived discrimination based on sexual orientation. Journal of Youth and Adolescence, 38(7), 1001–1014. 10.1007/s10964-009-9397-9 19636742 PMC3707280

[aur2162-bib-0003] American Psychiatric Association . (2013). Diagnostic and statistical manual of mental disorders (5th ed.). Arlington, VA: American Psychiatric Publishing.

[aur2162-bib-0004] Arseneault, L. , Bowes, L. , & Shakoor, S. (2010). Bullying victimization in youths and mental health problems: ‘Much ado about nothing’? Psychological Medicine, 40(5), 717–729. 10.1017/S0033291709991383 19785920

[aur2162-bib-0005] Asselmann, E. , Wittchen, H.‐U. , Lieb, R. , Höfler, M. , & Beesdo‐Baum, K. (2015). Danger and loss events and the incidence of anxiety and depressive disorders: A prospective‐longitudinal community study of adolescents and young adults. Psychological Medicine, 45(01), 153–163. 25065411 10.1017/S0033291714001160

[aur2162-bib-0006] Baron‐Cohen, S. , Wheelwright, S. , Skinner, R. , Martin, J. , & Clubley, E. (2001). The autism‐spectrum quotient (AQ): Evidence from Asperger syndrome/high‐functioning autism, males and females, scientists and mathematicians. Journal of Autism and Developmental Disorders, 31(1), 5–17. 11439754 10.1023/a:1005653411471

[aur2162-bib-0007] Boulter, C. , Freeston, M. , South, M. , & Rodgers, J. (2014). Intolerance of uncertainty as a framework for understanding anxiety in children and adolescents with autism spectrum disorders. Journal of Autism and Developmental Disorders, 44(6), 1391–1402. 24272526 10.1007/s10803-013-2001-x

[aur2162-bib-0008] Brown‐Lavoie, S. M. , Viecili, M. A. , & Weiss, J. A. (2014). Sexual knowledge and victimization in adults with autism spectrum disorders. Journal of Autism and Developmental Disorders, 44(9), 2185–2196. 10.1007/s10803-014-2093-y 24664634 PMC4131130

[aur2162-bib-0009] Bruggink, A. , Huisman, S. , Vuijk, R. , Kraaij, V. , & Garnefski, N. (2016). Cognitive emotion regulation, anxiety and depression in adults with autism spectrum disorder. Research in Autism Spectrum Disorder, 22, 34–44.

[aur2162-bib-0010] Cai, R. Y. , Richdale, A. L. , Dissanayake, C. , & Uljarević, M. (2018). Brief report: Inter‐relationship between emotion regulation, intolerance of uncertainty, anxiety, and depression in youth with autism spectrum disorder. Journal of Autism and Developmental Disorders, 48(1), 316–325. 10.1007/s10803-017-3318-7 28980172

[aur2162-bib-0011] Cappadocia, M. C. , Weiss, J. A. , & Pepler, D. (2012). Bullying experiences among children and youth with autism spectrum disorders. Journal of Autism and Developmental Disorders, 42(2), 266–277. 10.1007/s10803-011-1241-x 21499672

[aur2162-bib-0012] Cassidy, S. , Bradley, P. , Robinson, J. , Allison, C. , McHugh, M. , & Baron‐Cohen, S. (2014). Suicidal ideation and suicide plans or attempts in adults with Asperger's syndrome attending a specialist diagnostic clinic: a clinical cohort study. The Lancet Psychiatry, 1(2), 142–147. 26360578 10.1016/S2215-0366(14)70248-2

[aur2162-bib-0013] Charman, T. , Pickles, A. , Simonoff, E. , Chandler, S. , Loucas, T. , & Baird, G. (2011). IQ in children with autism spectrum disorders: Data from the Special Needs and Autism Project (SNAP). Psychological Medicine, 41(3), 619–627. 10.1017/S0033291710000991 21272389

[aur2162-bib-0014] Churchard, A. , Ryder, M. , Greenhill, A. , & Mandy, W. (2019). The prevalence of autistic traits in a homeless population. Autism, 23(3), 1362361318768484 10.1177/1362361318768484 29633853

[aur2162-bib-0015] Crane, L. , Maras, K. L. , Hawken, T. , Mulcahy, S. , & Memon, A. (2016). Experiences of autism spectrum disorder and policing in England and Wales: Surveying police and the autism community. Journal of Autism and Developmental Disorders, 46(6), 2028–2041. 10.1007/s10803-016-2729-1 26861714

[aur2162-bib-0016] Diener, E. , Emmons, R. A. , Larsen, R. J. , & Griffin, S. (1985). The satisfaction with life scale. Journal of Personality Assessment, 49(1), 71–75. 16367493 10.1207/s15327752jpa4901_13

[aur2162-bib-0017] Dinkler, L. , Lundström, S. , Gajwani, R. , Lichtenstein, P. , Gillberg, C. , & Minnis, H. (2017). Maltreatment‐associated neurodevelopmental disorders: A co‐twin control analysis. Journal of Child Psychology and Psychiatry, 58, 691–701. 10.1111/jcpp.12682 28094432

[aur2162-bib-0018] Eussen, M. L. , Van Gool, A. R. , Verheij, F. , De Nijs, P. F. , Verhulst, F. C. , & Greaves‐Lord, K. (2013). The association of quality of social relations, symptom severity and intelligence with anxiety in children with autism spectrum disorders. Autism, 17(6), 723–735. 22917843 10.1177/1362361312453882

[aur2162-bib-0019] Fisher, M. H. , Moskowitz, A. L. , & Hodapp, R. M. (2012). Vulnerability and experiences related to social victimization among individuals with intellectual and developmental disabilities. Journal of Mental Health Research in Intellectual Disabilities, 5(1), 32–48. 10.1080/19315864.2011.592239

[aur2162-bib-0020] Fisher, M. H. , Moskowitz, A. L. , & Hodapp, R. M. (2013). Differences in social vulnerability among individuals with autism spectrum disorder, Williams Syndrome, and down syndrome. Research in Autism Spectrum Disorder, 7(8), 931–937. 10.1016/j.rasd.2013.04.009 PMC3670772 23745132

[aur2162-bib-0021] Gillian, D. , Lynn, G. M. , Kenneth, G. , Michael, L. , & Priscilla, H. (2017). Researching the financial abuse of individuals lacking mental capacity. The Journal of Adult Protection, 19(6), 394–405. 10.1108/JAP-05-2017-0022

[aur2162-bib-0022] Greenberg, D. M. , Warrier, V. , Allison, C. , & Baron‐Cohen, S. (2018). Testing the Empathizing–Systemizing theory of sex differences and the Extreme Male Brain theory of autism in half a million people. Proceedings of the National Academy of Sciences, 115(48), 12152–12157. 10.1073/pnas.1811032115 PMC6275492 30420503

[aur2162-bib-0023] Hayes, A. F. (2012). PROCESS: A versatile computational tool for observed variable mediation, moderation, and conditional process modeling. Kansas: University of Kansas.

[aur2162-bib-0024] Hofvander, B. , Delorme, R. , Chaste, P. , Nyden, A. , Wentz, E. , Stahlberg, O. , … Leboyer, M. (2009). Psychiatric and psychosocial problems in adults with normal‐intelligence autism spectrum disorders. BMC Psychiatry, 9, 35 10.1186/1471-244X-9-35 19515234 PMC2705351

[aur2162-bib-0025] Hollocks, M. J. , Jones, C. R. , Pickles, A. , Baird, G. , Happé, F. , Charman, T. , & Simonoff, E. (2014). The association between social cognition and executive functioning and symptoms of anxiety and depression in adolescents with autism spectrum disorders. Autism Research, 7(2), 216–228. 24737743 10.1002/aur.1361

[aur2162-bib-0026] Howlin, P. (2013). Social disadvantage and exclusion: adults with autism lag far behind in employment prospects. Journal of the American Academy of Child & Adolescent Psychiatry, 52(9), 897–899. 23972691 10.1016/j.jaac.2013.06.010

[aur2162-bib-0027] Jawaid, A. , Riby, D. M. , Owens, J. , White, S. W. , Tarar, T. , & Schulz, P. E. (2012). 'Too withdrawn' or 'too friendly': considering social vulnerability in two neuro‐developmental disorders. Journal of Intellectual Disability Research, 56(4), 335–350. 10.1111/j.1365-2788.2011.01452.x 21801261

[aur2162-bib-0028] Jenkins, R. , Bhugra, D. , Bebbington, P. , Brugha, T. , Farrell, M. , Coid, J. , … Meltzer, H. (2008). Debt, income and mental disorder in the general population. Psychological Medicine, 38(10), 1485–1493. 18184442 10.1017/S0033291707002516

[aur2162-bib-0029] Joshi, G. , Wozniak, J. , Petty, C. , Martelon, M. K. , Fried, R. , Bolfek, A. , … Biederman, J. (2013). Psychiatric comorbidity and functioning in a clinically referred population of adults with autism spectrum disorders: A comparative study. Journal of Autism and Developmental Disorders, 43(6), 1314–1325. 10.1007/s10803-012-1679-5 23076506

[aur2162-bib-0030] Kirchner, J. , Ruch, W. , & Dziobek, I. (2016). Brief report: Character strengths in adults with autism spectrum disorder without intellectual impairment. Journal of Autism and Developmental Disorders, 46(10), 3330–3337. 27457365 10.1007/s10803-016-2865-7

[aur2162-bib-0031] Kroenke, K. , Spitzer, R. L. , & Williams, J. B. W. (2001). The PHQ‐9: Validity of a brief depression severity measure. Journal of General Internal Medicine, 16(9), 606–613. 10.1046/j.1525-1497.2001.016009606.x 11556941 PMC1495268

[aur2162-bib-0032] Lauren, B.‐F. , Carla, A. M. , & Shaun, M. E. (2017). The combined impact of social support and perceived stress on quality of life in adults with autism spectrum disorder and without intellectual disability. Autism, 22, 1362361317703090–1362361317703711. 10.1177/1362361317703090 PMC5711618 28666391

[aur2162-bib-0033] Lindert, J. , von Ehrenstein, O. S. , Grashow, R. , Gal, G. , Braehler, E. , & Weisskopf, M. G. (2014). Sexual and physical abuse in childhood is associated with depression and anxiety over the life course: Systematic review and meta‐analysis. International Journal of Public Health, 59(2), 359–372. 24122075 10.1007/s00038-013-0519-5

[aur2162-bib-0034] Lorenc, T. , Rodgers, M. , Rees, R. , Wright, K. , Melton, H. , & Sowden, A. (2016). Preventative co‐ordinated low‐level support for adults with high‐functioning autism: Systematic review and service mapping. London: University College London.

[aur2162-bib-0035] Lugnegard, T. , Hallerback, M. U. , & Gillberg, C. (2011). Psychiatric comorbidity in young adults with a clinical diagnosis of Asperger syndrome. Research in Developmental Disabilities, 32(5), 1910–1917. 10.1016/j.ridd.2011.03.025 21515028

[aur2162-bib-0036] Mazurek, M. O. (2013). Loneliness, friendship, and well‐being in adults with autism spectrum disorders. Autism, 18(3), 223–232. 10.1177/1362361312474121 24092838

[aur2162-bib-0037] Nicolaidis, C. , Raymaker, D. , McDonald, K. , Dern, S. , Ashkenazy, E. , Boisclair, C. , … Baggs, A. (2011). Collaboration strategies in nontraditional community‐based participatory research partnerships: Lessons from an academic‐community partnership with autistic self‐advocates. Progress in Community Health Partnerships, 5(2), 143–150. 10.1353/cpr.2011.0022 21623016 PMC3319698

[aur2162-bib-0038] Paul, K. I. , & Moser, K. (2009). Unemployment impairs mental health: Meta‐analyses. Journal of Vocational Behavior, 74(3), 264–282.

[aur2162-bib-0039] Preacher, K. J. , & Hayes, A. F. (2008). Asymptotic and resampling strategies for assessing and comparing indirect effects in multiple mediator models. Behavior Research Methods, 40(3), 879–891. 18697684 10.3758/brm.40.3.879

[aur2162-bib-0040] Rava, J. , Shattuck, P. , Rast, J. , & Roux, A. (2017). The prevalence and correlates of involvement in the criminal justice system among youth on the autism spectrum. Journal of Autism and Developmental Disorders, 47(2), 340–346. 10.1007/s10803-016-2958-3 27844248

[aur2162-bib-0041] Roy, M. , Prox‐Vagedes, V. , Ohlmeier, M. D. , & Dillo, W. (2015). Beyond childhood: Psychiatric comorbidities and social background of adults with Asperger syndrome. Psychiatria Danubina, 27(1), 50–59. 25751431

[aur2162-bib-0042] Ruzich, E. , Allison, C. , Smith, P. , Watson, P. , Auyeung, B. , Ring, H. , & Baron‐Cohen, S. (2015). Measuring autistic traits in the general population: A systematic review of the Autism‐Spectrum Quotient (AQ) in a nonclinical population sample of 6,900 typical adult males and females. Molecular Autism, 6(1), 2. 25874074 10.1186/2040-2392-6-2 PMC4396128

[aur2162-bib-0043] Schmidt, L. , Kirchner, J. , Strunz, S. , Brozus, J. , Ritter, K. , Roepke, S. , & Dziobek, I. (2015). Psychosocial functioning and life satisfaction in adults with autism spectrum disorder without intellectual impairment. Journal of Clinical Psychology, 71(12), 1259–1268. 10.1002/jclp.22225 26406481

[aur2162-bib-0044] Sharpe, D. L. , & Baker, D. L. (2007). Financial issues associated with having a child with autism. Journal of Family and Economic Issues, 28(2), 247–264. 10.1007/s10834-007-9059-6

[aur2162-bib-0045] Spitzer, R. L. , Kroenke, K. , Williams, J. B. , & Lowe, B. (2006). A brief measure for assessing generalized anxiety disorder: The GAD‐7. Archives of Internal Medicine, 166(10), 1092–1097. 10.1001/archinte.166.10.1092 16717171

[aur2162-bib-0046] Taylor, J. L. , & Gotham, K. O. (2016). Cumulative life events, traumatic experiences, and psychiatric symptomatology in transition‐aged youth with autism spectrum disorder. Journal of Neurodevelopmental Disorders, 8, 28 10.1186/s11689-016-9160-y 27468315 PMC4962443

[aur2162-bib-0047] Taylor, J. L. , Henninger, N. A. , & Mailick, M. R. (2015). Longitudinal patterns of employment and postsecondary education for adults with autism and average‐range IQ. Autism, 19(7), 785–793. 10.1177/1362361315585643 26019306 PMC4581899

[aur2162-bib-0048] Wigham, S. , Rodgers, J. , South, M. , McConachie, H. , & Freeston, M. (2015). The interplay between sensory processing abnormalities, intolerance of uncertainty, anxiety and restricted and repetitive behaviours in autism spectrum disorder. Journal of Autism and Developmental Disorders, 45(4), 943–952. 25261248 10.1007/s10803-014-2248-x

