# Supplementary material for: The Vulnerability Experiences Quotient (VEQ): A Study of Vulnerability, Mental Health and Life Satisfaction in Autistic Adults
Source: Autism Res. 2019 Jul 5;12(10):1516–28. doi: 10.1002/aur.2162 (PMC6851759; doi:10.1002/aur.2162)
Supplement: Supplementary file 1 — Appendix S1: Supporting Information. [file AUR-12-1516-s001.doc]

3/29/2017 Qualtrics Survey Software


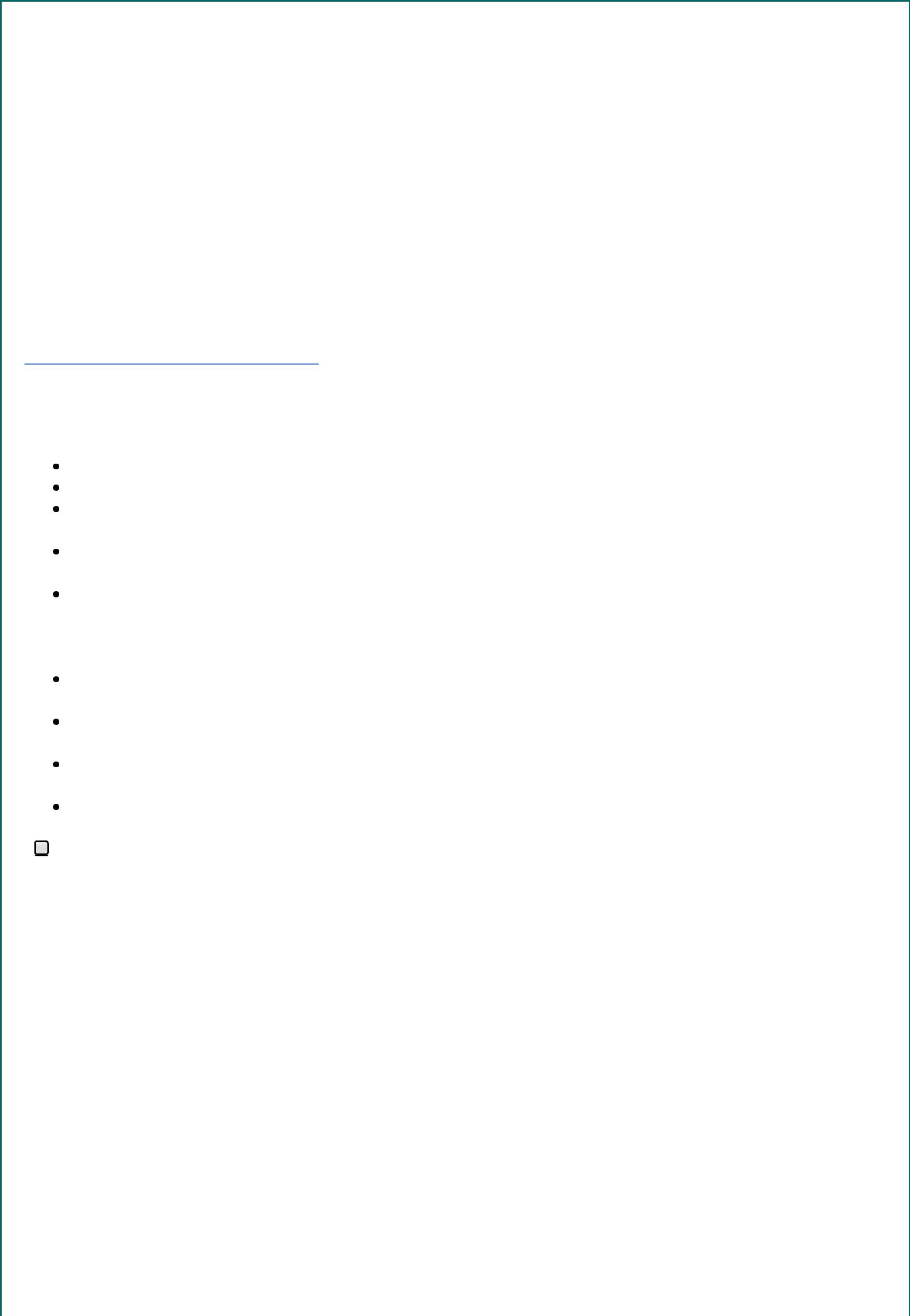


Welcome and consent

Welcome to the autism and vulnerability survey

This survey is part of ongoing research at the Autism Research Centre at the University of Cambridge about life experiences and mental health in individuals with and without autism.

Before you decide whether to take part, it is important for you to understand why this research is being done and what it will involve. Please take time to read the Study Information Sheet carefully and discuss it with others if you wish.

You can contact the research team on autism­vulnerablity@medschl.cam.ac.uk

[Click here to open the study information sheet](https://eu.qualtrics.com/ControlPanel/File.php?F=F_3CtmXMniMDTzgQR&download=1)

Please read each statement below and check the box at the bottom to accept.

I am over 18 years old

I have read and understood the study information sheet

I have had the opportunity to ask questions by emailing autism­ vulnerability@medschl.cam.ac.uk

I understand that all information I provide will be stored securely in compliance with the Data Protection Act and the Freedom of Information Act

I understand that any personally identifying information I provide will remain completely confidential and will not be shared outside of the research team (*except as might be required by* *law*). Please note that we have a responsibility to inform the relevant authorities if we receive information which would allow the identification of a person who is at risk of harm.

I understand that anonymous data gathered in this study may be shared with other researchers and used for future research

I understand that my participation is voluntary and that I am free to withdraw at any time without giving a reason

I understand that some of the free text answers may be used as quotes in journal papers and at conferences

I understand that certain questions might be upsetting in nature

I agree with the above statements and consent to take part in this study

The following survey includes some questions that are sensitive and potentially upsetting in nature. Please remember that all of your responses are anonymous (we will not be able to contact you or identify you from your responses). You can stop the survey at anytime if you feel at all anxious or uncomfortable.

If you are concerned about your current mental health, please contact your family doctor (GP if you are in the UK). If you think that you or someone else is in immediate danger, please contact the emergency services (e.g. call 999 if you are in the UK). Please also consider contacting one of the organisations below if you require support relating to particular issues covered in the survey.

Mental health

SANE offers specialist emotional support and information to anyone affected by mental illness. Website: www.sane.org.uk

Helpline: 0300 304 7000 (Everyday 4:30pm to 10:30pm)

Mind provide information and advice about self­harm and mental health. Website: www.mind.org.uk Mind Infoline: 0300 123 3393 (weekdays 9am ­ 6pm). Services are also available for people with hearing and speech difficulties, and an interpreting service using Language Line is provided.

Harmless provide information and support to people who self­harm as well as those supporting them: www.harmless.org.uk

Samaritans 24­hour free confidential support to anyone in crisis. Website: www.samaritans.org Call on: 116 123, Email on:

jo@samaritans.org.

https://eu.qualtrics.com/ControlPanel/Ajax.php?action=GetSurveyPrintPreview 1/12

3/29/2017 Qualtrics Survey Software

Papyrus provide confidential advice and support to young people (up to age 35) and anyone worried about a young person. Website: www.papyrus­uk.org HOPELineUK: 0800 068 41 41, Email: pat@papyrus­uk.org, SMS: 07786 209697


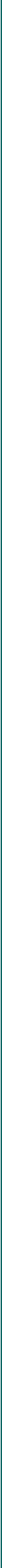

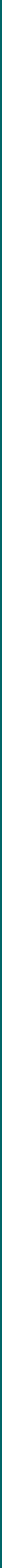


A list of relevant support services outside of the UK can be found through the Befrienders website:

http://www.befrienders.org/

Abuse

The National Association for People Abused in Childhood (NAPAC) provides a free support line for adults who have suffered any type of abuse in childhood. Website: www.napac.org.uk. Helpline: 0808 801 0331 (Monday­Thursday 10:00am­ 9.00pm and Friday 10.00am­6.00pm)

Mothers of Sexually Abused Children (MOSAC) Supporting all non­abusing parents and carers whose children have been sexually abused. We provide various types of support services and information for parents, carers and professionals dealing with child sexual abuse. Website: www.mosac.org.uk. Helpline: 0800 980 1958

Refuge run a helpline and provide refuge for people affected by domestic violence and abuse. Website: www.refuge.org.uk

Helpline: 0808 2000 247. Services are also available for people with hearing and speech difficulties, and an interpreting service using Language Line is provided.

Bullybusters free phone helpline providing support and advice for victims of bullying and their families. Also operates a website with advice for young people, parents and carers and professionals and a message board for young people. Call on:

0800 169 6928 Website: www.bullybusters.org.uk.

For a list of relevant support services outside the UK please see: www.crin.org/en/library/publications/childrens­helplines­ worldwide and www.hotpeachpages.net/a/countries.html

Autism

National Autistic Society provides information and advice for people with autism spectrum conditions and their families. Helpline: 0808 800 4104 (Monday­Thursday 10am ­ 4pm Friday 9am ­ 3pm). The NAS website www.autism.org.uk has comprehensive information on a wide range of topics including; diagnosis, education, employment, benefits, managing money, contact with the criminal justice system, health care, mental health and advocacy.

The National Autistic Society is a UK based organisation. To find relevant support and advice if you live outside the UK please see: www.autism.org.uk/services/helplines/outside­uk/round­world.aspx

This survey will take 30 ­ 40 minutes to complete. It has 4 parts.

Section 1 "About you" asks 24 background questions about you and any diagnoses you may have of mental health and developmental conditions (5­10 minutes).

Section 2 "Your thoughts and feelings" asks 21 questions about your current mood, and feelings about your life (5­10 minutes).

Section 3 "Your life experiences" asks about life experiences relating to different areas of life (10­15 minutes).

Section 4 "Your personality" asks 10 questions about your personality (3­5 minutes).

You can have a break at any point and come back later to complete the remaining questions. Your responses will be saved automatically so when you click on the link again you will come back to the same place in the survey. The survey will only save responses from complete pages, so make sure you finish the current page and move on to the next page, before closing the survey.

You must come back to the survey on the same computer otherwise your answers will not be available.

Demographics

Section 1 About you

https://eu.qualtrics.com/ControlPanel/Ajax.php?action=GetSurveyPrintPreview 2/12

3/29/2017 Qualtrics Survey Software


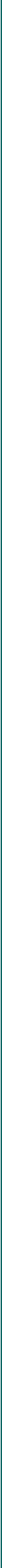

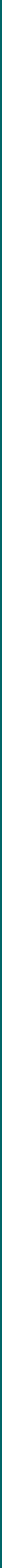


In which country do you currently live?


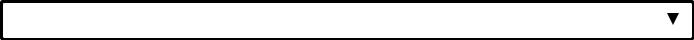


What is your date of birth? Please enter as dd/mm/yyyy


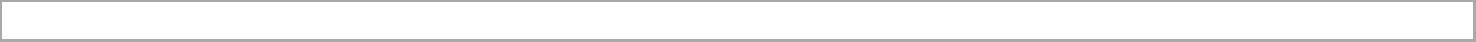


Which gender was assigned to you at birth?

Male


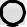


Female


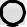


Other


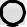


Which gender do you currently identify with?

Male


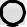


Female


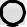


Non­binary


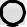


Other


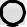


Have you ever been in paid employment?

Yes


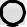


No


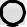


What is your current employment status? (please tick all that apply)

In full time paid employment


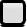


In part time paid employment


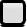


Voluntary employment


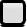


Student


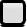


Self­employed


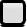


Seeking work


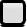


Unable to work


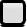


Carer or homemaker


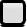


Retired


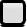


Have you ever been in a relationship (e.g. had a girlfriend or boyfriend for more than a month)?

Yes


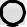


No


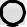


https://eu.qualtrics.com/ControlPanel/Ajax.php?action=GetSurveyPrintPreview 3/12

3/29/2017 Qualtrics Survey Software


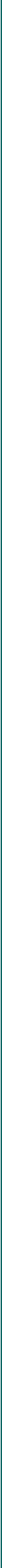

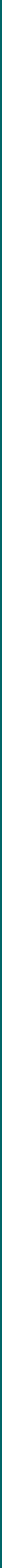


What is your current relationship status? (Please tick all that apply)

Single


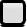


Married


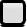


In a civil partnership


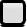


Cohabiting


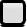


In a long term relationship (not cohabiting)


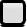


Divorced


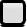


Widowed


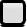


Separated


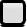


Do you have children?

Yes


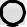


No


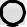


How old where you when you left full time education?


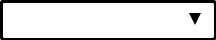


Have you ever been to university?

Yes


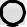


No


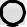


What is your highest level of qualification?

No formal qualifications


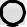


High school/secondary school level qualifications


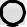


Further vocational qualifications


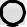


University undergraduate level qualification (BA, BSc etc.)


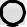


University post­graduate level qualification (MA, MSc, PhD, Certificate, etc.)


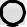


Did you ever attend a school for children with special educational needs?

Yes


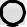


No


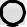


Did you ever receive any additional help at school, such as having a teaching assistant who worked with just you in the classroom?

Yes


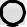


No


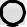


https://eu.qualtrics.com/ControlPanel/Ajax.php?action=GetSurveyPrintPreview 4/12

3/29/2017 Qualtrics Survey Software


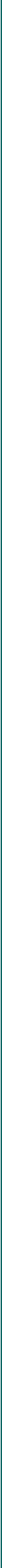

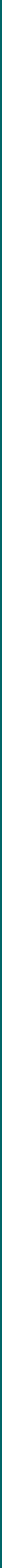


Who do you currently live with? (Please tick all that apply)

No one, I live on my own


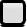


My parent(s) or guardian(s)


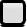


Other family member(s)


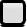


Friend(s)


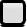


Other resident(s) of shared accommodation


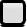


My partner


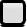


My child/children


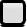


Does anyone regularly come and support you with household activities such as cooking, cleaning, shopping, washing or organising bills?

Yes


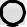


No


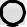


Have you ever been diagnosed with any of the following conditions by a clinician?

Please tick all that apply.


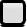
 Alcohol or other drug abuse or addiction
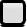
 Anxiety disorder

Attentional deficit hyperactivity disorder (ADHD)/Attentional deficit disorder (ADD)


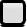


Autism spectrum condition (Asperger Syndrome/Autism/PDD­NOS)


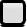


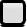
 Bipolar disorder


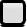
 Conduct disorder


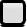
 Depression


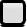
 Dyslexia


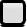
 Dyspraxia/Developmental coordination disorder


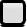
 Eating disorder


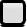
 Intellectual disability


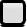
 Generalised anxiety disorder


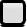
 Language delay


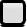
 Obsessive compulsive disorder (OCD)


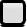
 Oppositional defiant disorder (ODD)


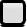
 Panic disorder


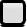
 Personality disorder


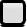
 Post­traumatic stress disorder


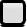
 Schizophrenia/Psychosis


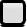
 Sensory processing disorder


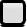
 Social phobia/Social anxiety disorder


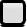
 Specific phobia


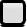
 Tourette syndrome/Tic disorder

Do you suspect that you have any condition that you have not been diagnosed with?

Yes


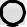


No


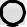


Which condition(s) do you suspect that you have (or have had)?

Please tick all that apply.

https://eu.qualtrics.com/ControlPanel/Ajax.php?action=GetSurveyPrintPreview 5/12

3/29/2017


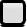
 Alcohol or other drug abuse or addiction
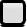
 Anxiety Disorder


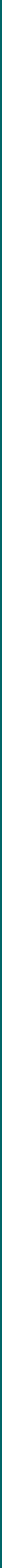


Attentional deficit hyperactivity disorder (ADHD)/Attentional deficit disorder (ADD)


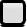


Autism spectrum condition (Asperger Syndrome/Autism/PDD­NOS)


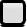


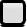
 Bipolar disorder


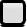
 Conduct disorder


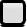
 Depression


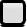
 Dyslexia


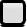
 Dyspraxia/Developmental coordination disorder


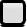
 Eating disorder


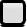
 Intellectual disability


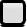
 Generalised anxiety disorder

Qualtrics Survey Software

Language delay

Obsessive compulsive disorder (OCD)

Oppositional defiant disorder (ODD)

Panic disorder

Personality disorder

Post­traumatic stress disorder

Schizophrenia/Psychosis

Sensory processing disorder

Social phobia/Social anxiety disorder

Specific phobia

Tourette syndrome/Tic disorder

Have you ever been diagnosed with any other mental health or developmental condition(s)?

Yes

No

Please state your diagnosis.

How old were you when you were diagnosed with an autism spectrum condition?

Who gave you this diagnosis?

Paediatricians

Psychiatrist

Neurologist

Clinical psychologist

Educational psychologist

Self­diagnosis

Not sure

Other. Please state the type of professional who gave the diagnosis

Does anyone in your family have a diagnosis of an autism spectrum condition?

Yes

https://eu.qualtrics.com/ControlPanel/Ajax.php?action=GetSurveyPrintPreview 6/12

3/29/2017 Qualtrics Survey Software

No

Which member(s) of your family have a diagnosis of an autism spectrum condition? Please tick all that apply.

My child

My parent

My grandparent

My brother and/or sister

My aunt and/or uncle

My cousin

Other

Depression, anxiety and life satisfaction

Section 2 Your thoughts and feelings

Below are five statements that you may agree or disagree with. Using the 1 ­ 7 scale below, indicate your agreement with each item.

Neither

Strongly Slightly agree nor Slightly Strongly

agree Agree agree disagree disagree Disagree disagree

In most ways my life is

close to my ideal.

The conditions of my life are

excellent.

I am satisfied with my life.

So far I have got the

important things I want in

life.

If I could live my life over, I

would change almost

nothing.

Over the last 2 weeks, on how many days have you been bothered by any of the following problems?

More than half

Not at all Several days the days Nearly everyday

Little interest or pleasure in doing things

Feeling down, depressed or hopeless

Trouble falling or staying asleep, or sleeping too

much

Feeling tired or having little energy

Poor appetite or overeating

Feeling bad about yourself – or that you are a

failure or have let yourself or your family down

Trouble concentrating on things, such as reading

the newspaper or watching television

https://eu.qualtrics.com/ControlPanel/Ajax.php?action=GetSurveyPrintPreview 7/12

3/29/2017 Qualtrics Survey Software

More than half

Not at all Several days the days Nearly everyday

Moving or speaking so slowly that other people

could have noticed, or the opposite – being so

fidgety or restless that you have been moving

around a lot more than usual

Thoughts that you would be better off dead or of

hurting yourself in some way

Feeling nervous, anxious or on edge

Not being able to stop or control worrying

Worrying too much about different things

Trouble relaxing

Being so restless it is hard to sit still

Becoming easily annoyed or irritable

Feeling afraid as if something awful might happen

Vulnerability questions

Section 3 Your life experiences

The next section includes questions about different kinds of life experiences. Some of these experiences may be upsetting to remember, and there will be mention of suicide, self harm and abuse.

I am prepared to continue

I would like to skip this section

Below is a list of statements about different life experiences. Please choose ‘Agree’, ‘Disagree’ or ‘No opportunity’ for each statement.

Select ‘No opportunity’ if you feel the statement is not relevant to your life, for example, if you have never been employed you would select “no opportunity” for statements referring to work.

Agree Disagree No opportunity

I left a school/college/university without a qualification

because I failed my exams

I have been bullied by someone in my family

As a child, other children bullied me

I have had possessions forcibly removed by debt

collectors

There was a period of my life where I was regularly

using alcohol or another (non­prescribed) drug in order to

cope.

I was signed off from work for at least 2 months due to

anxiety, depression or any other mental health reason

I spent at least a year unemployed and seeking work

I was incorrectly diagnosed with a mental health

condition (e.g. ADHD instead of autism)

I have been pressured into sexual activity

My child/ren were subject to a child protection

investigation due to concerns about my ability to care for

them

https://eu.qualtrics.com/ControlPanel/Ajax.php?action=GetSurveyPrintPreview 8/12

3/29/2017 Qualtrics Survey Software

Agree Disagree No opportunity

I have been bullied by someone at work

There has always been someone in my life who would

try to help me if I was in trouble

My partner forced me into sexual activity

There has been a period in my life where I did not have

enough money to meet my basic needs (e.g. food, rent,

medical care)

I was temporarily or permanently excluded from

school/college/university

I have a criminal record

My child/ren were referred to social services due to

concerns about my ability to care for them

There has always been someone in my life who would

care for me if I was ill

I have been sectioned because of a mental health

condition

As a child, an adult hurt me badly enough that it left

marks on my body

I was charged with a criminal offense (not including

speeding or parking fines)

I have been tricked or pressured into breaking the law

I was sacked from a job

My partner physically hurt me e.g. shoved, slapped or

punched me

I missed more than 4 weeks of school/college/university

due to anxiety, depression or any other mental health

reason

Disciplinary action was taken against me at work

As a child, other children left me out of activities

I lost custody of my child/ren through court proceedings

due to concerns about my ability to care for them

As a child, children spread rumours about me or talked

about me behind my back

I left a job because I was unable to deal with the work

environment and/or the demands of the job

My parents/carers tried to get additional support for me

at school but the school did not provide any

I was cautioned by the police (not including cautions for

minor traffic offences)

An educational, medical or social work professional

questioned my ability to care for my child

I have been physically forced into sexual activity

There has been a period in my life where I had debts

(other than a mortgage or student loan) that were greater

than my yearly income

I spent time in prison or a juvenile detention centre

I have been regularly overlooked for promotions or

contract renewals at work

My partner threatened to harm me or to harm someone I

care about

As a child, another child hurt me badly enough that it left

marks on my body (e.g bruises or scratches)

I have had a mental health condition that affected my

daily life

As an adult, I have been hurt by someone badly enough

that it left marks on my body (e.g bruises or scratches)

https://eu.qualtrics.com/ControlPanel/Ajax.php?action=GetSurveyPrintPreview 9/12

3/29/2017 Qualtrics Survey Software

Agree Disagree No opportunity

I have made suicide plans

I left a job because I was being treated badly by

colleagues

I dropped out of school/college/university

I was arrested by the police

I had to leave my home because I was unable to keep

up with mortgage or rent payments

As a child, children called me names or insulted me

I have been bullied by someone that I considered to be a

friend

I have been unable to get a job which matches my level

of training and qualification

As a child, an adult humiliated, embarrassed or scared

me

I avoided attending lessons or lectures at

school/college/university because I found them stressful

I have attempted suicide

I have been tricked or pressured in to giving someone

money or possessions

My partner took advantage of me financially

As a child, an adult touched me in a sexual way, or tried

to make me touch them in a sexual way

There was a period in my life where I had nowhere safe

to live

As a child, an adult swore at me or called me names like

stupid, ugly or lazy

I have always known that there is someone in my life

who loves me

My partner humiliated or embarrassed me

I have deliberately harmed myself

Please use the box below to describe any other experiences you have had that are relevant to your well being and mental health.

AQ­10

Section 4 Your personality

Please indicate how much you agree with the following statements:

https://eu.qualtrics.com/ControlPanel/Ajax.php?action=GetSurveyPrintPreview 10/12

3/29/2017 Qualtrics Survey Software

Slightly Definitely

Definitely Agree Slightly Agree Disagree Disagree

I often notice small sounds when others do not

I usually concentrate more on the whole picture,

rather than the small details

I find it easy to do more than one thing at once

If there is an interruption, I can switch back to

what I was doing very quickly

I find it easy to ‘read between the lines’ when

someone is talking to me

I know how to tell if someone listening to me is

getting bored

When I’m reading a story I find it difficult to

work out the characters’ intentions

I like to collect information about categories of

things (e.g. types of car, types of bird, types of

train, types of plant etc)

I find it easy to work out what someone is

thinking or feeling just by looking at their face

I find it difficult to work out people’s intentions

Goodbye and support

Thank you very much for taking part in this survey.

The results of the survey will help improve our understanding of the life experiences of adults with and without autism. We hope that these findings will help improve mental health and social care services, and help autistic adults to lead healthier, happier lives.

If you have any questions about this project or need any more information, please contact the research team by emailing: autism­vulnerablity@medschl.cam.ac.uk

Would you like to leave your email address so you can be sent information about the results of the survey? Note that your email address will be stored separately to your survey responses.

Yes

No

If you are concerned about your current mental health, please contact your family doctor (GP if you are in the UK). If you think that you or someone else is in immediate danger, please contact the emergency services (e.g. call 999 if you are in the UK). Please also consider contacting one of the organisations below if you require support relating to particular issues covered in the survey.

Mental health

SANE offers specialist emotional support and information to anyone affected by mental illness.

Website: www.sane.org.uk Helpline: 0300 304 7000 (Everyday 4:30pm to 10:30pm)

Mind provide information and advice about self­harm and mental health. Website: www.mind.org.uk Mind Infoline: 0300 123 3393 (weekdays 9am ­ 6pm). Services are also available for people with hearing and speech difficulties, and an interpreting service using Language Line is provided.

Harmless provide information and support to people who self­harm as well as those supporting them: www.harmless.org.uk

https://eu.qualtrics.com/ControlPanel/Ajax.php?action=GetSurveyPrintPreview 11/12

3/29/2017 Qualtrics Survey Software

Samaritans 24­hour free confidential support to anyone in crisis. Website: www.samaritans.org Call on: 116 123, Email on:

jo@samaritans.org.

Papyrus provide confidential advice and support to young people (up to age 35) and anyone worried about a young person. Website: www.papyrus­uk.org HOPELineUK: 0800 068 41 41, Email: pat@papyrus­uk.org, SMS: 07786 209697

A list of relevant support services outside of the UK can be found through the Befrienders website: http://www.befrienders.org/

Abuse

The National Association for People Abused in Childhood (NAPAC) provides a free support line for adults who have suffered any type of abuse in childhood. Website: www.napac.org.uk. Helpline: 0808 801 0331 (Monday­Thursday 10:00am­ 9.00pm and Friday 10.00am­6.00pm)

Mothers of Sexually Abused Children (MOSAC) Supporting all non­abusing parents and carers whose children have been sexually abused. We provide various types of support services and information for parents, carers and professionals dealing with child sexual abuse. Website: www.mosac.org.uk. Helpline: 0800 980 1958

Refuge run a helpline and provide refuge for people affected by domestic violence and abuse. Website: www.refuge.org.uk

Helpline: 0808 2000 247. Services are also available for people with hearing and speech difficulties, and an interpreting service using Language Line is provided.

Bullybusters free phone helpline providing support and advice for victims of bullying and their families. Also operates a website with advice for young people, parents and carers and professionals and a message board for young people. Call on:

0800 169 6928 Website: www.bullybusters.org.uk.

For a list of relevant support services outside the UK please see: www.crin.org/en/library/publications/childrens­helplines­ worldwide and www.hotpeachpages.net/a/countries.html

Autism

National Autistic Society provides information and advice for people with autism spectrum conditions and their families. Helpline: 0808 800 4104 (Monday­Thursday 10am ­ 4pm Friday 9am ­ 3pm). The NAS website www.autism.org.uk has comprehensive information on a wide range of topics including; diagnosis, education, employment, benefits, managing money, contact with the criminal justice system, healthcare, mental health and advocacy.

The National Autistic Society is a UK based organisation. To find relevant support and advice if you live outside the UK please see: www.autism.org.uk/services/helplines/outside­uk/round­world.aspx

https://eu.qualtrics.com/ControlPanel/Ajax.php?action=GetSurveyPrintPreview 12/12
