# Supplementary material for: The Vulnerability Experiences Quotient (VEQ): A Study of Vulnerability, Mental Health and Life Satisfaction in Autistic Adults
Source: Autism Res. 2019 Jul 5;12(10):1516–28. doi: 10.1002/aur.2162 (PMC6851759; doi:10.1002/aur.2162)
Supplement: Supplementary file 2 — Table S1: Logistical Regression Analyses for Each Item in the VEQ Adjusted for Age and Sex. Excludes participants who did not report their age (n = 3) and those whose gender was transgender/non‐binary/other (n = 53). P values have been adjusted for the 60 multiple comparisons using the Bonferroni correction. Items for which there was a significant group difference are in bold font. [file AUR-12-1516-s002.docx]

Supplementary Table 1. Logistical regression analyses for each item in the VEQ adjusted for age and sex. Excludes participants who did not report their age (n = 3) and those whose gender was transgender/non-binary/other (n = 53). P values have been adjusted for the 60 multiple comparisons using the Bonferroni correction. Items for which there was a significant group difference are in **bold font**.

|  | Item | Autism | Control | χ² (Wald) |  | Adjusted odds ratio(95% CI) |
| --- | --- | --- | --- | --- | --- | --- |
| Education | **I dropped out of school/college/university** | 36% (135/374) | 19%  (50/264) | 19.01 | *p* < .001 | 2.40  (1.62-3.55) |
|  | **I missed more than 4 weeks of school/college/university due to anxiety or depression** | 44% (163/374) | 14%  (37/264) | 48.72 | *p* < .001 | 4.57  (2.98-7.00) |
|  | **I was temporarily or permanently excluded from school/college/university** | 13% (50/374) | 3%  (7/264) | 11.98 | *p* = .032 | 4.31  (1.88-9.85) |
|  | **My parents/carers tried to get additional support for me at school but the school did not provide any** | 19% (71/374) | 6%  (17/264) | 11.52 | *p* = .041 | 2.77  (1.54-4.98) |
|  | I left a school/college/university without a qualification because I failed my exams | 15% (55/374) | 8%  (21/264) | 3.467 | *p* = 1.00 | 1.70  (0.97-2.99) |
|  | **I avoided attending lessons or lectures at school/college/university because I found them stressful** | 51% (189/374) | 16%  (42/264) | 67.02 | *p* < .001 | 5.64  (3.73-8.52) |
| Employment | **I was signed off from work for at least 2 months due to anxiety, depression or any other mental health reason** | 42% (142/341) | 14%  (36/256) | 45.33 | *p* < .001 | 4.41  (2.86-6.79) |
|  | **I spent at least a year unemployed and seeking work** | 47% (160/341) | 15%  (38/256) | 48.76 | *p* < .001 | 4.47  (2.94-6.80) |
|  | **I was sacked from a job** | 41% (140/341) | 24%  (61/256) | 18.88 | *p* < .001 | 2.34  (1.60-3.44) |
|  | **Disciplinary action was taken against me at work** | 28% (95/341) | 14%  (35/256) | 13.44 | *p* = .015 | 2.33  (1.48-3.65) |
|  | **I left a job because I was unable to deal with the work environment and/or the demands of the job** | 72% (247/341) | 32%  (81/256) | 88.90 | *p* < .001 | 6.42  (4.36-9.45) |
|  | **I have been regularly overlooked for promotions or contract renewals at work** | 31% (106/341) | 11%  (30/256) | 25.08 | *p* < .001 | 3.29  (2.06-5.24) |
|  | **I left a job because I was being treated badly by colleagues** | 46% (158/341) | 19%  (207/256) | 25.10 | *p* < .001 | 3.29  (2.06-5.24) |
|  | **I have been unable to get a job which matches my level of training and qualification** | 53% (181/341) | 21%  (53/255) | 45.56 | *p* < .001 | 3.75  (2.56-5.51) |
| Finances | I have had possessions forcibly removed by debt collectors | 4%  (14/374) | 4%  (10/264) | 0.03 | *p* = 1.00 | 0.92  (0.38-2.22) |
|  | **There has been a period in my life where I did not have enough money to meet my basic needs (e·g· food, rent, medical care)** | 43%  (160/374) | 25%  (67/264) | 17.26 | *p* = .002 | 2.17  (1.50-3.12) |
|  | **There has been a period in my life where I had debts (other than a mortgage or student loan) that were greater than my yearly income** | 25%  (95/374) | 13%  (35/264) | 12.36 | *p* = .026 | 2.23  (1.43-3.50) |
|  | I had to leave my home because I was unable to keep up with mortgage or rent payments | 12%  (43/374) | 4%  (11/264) | 7.65 | *p* = .340 | 2.72  (1.34-5.53) |
|  | **There was a period in my life where I had nowhere safe to live** | 24%  (88/374) | 11%  (30/264) | 14.32 | *p* = .009 | 2.48  (1.55-3.98) |
| Social services | My child/ren were subject to a child protection investigation due to concerns about my ability to care for them | 9%  (12/139) | 2%  (3/167) | 5.60 | *p* = 1.00 | 4.91  (1.31-18.36) |
|  | My child/ren were referred to social services due to concerns about my ability to care for them | 14%  (20/139) | 1%  (1/167) | 5.60 | *p* = .081 | 28.16  (3.66-216.55) |
|  | I lost custody of my child/ren through court proceedings due to concerns about my ability to care for them | 4%  (6/139) | 1%  (1/167) | 2.99 | *p* = 1.00 | 6.77  (0.78-59.20) |
|  | **An educational, medical or social work professional questioned my ability to care for my child** | 19%  (26/139) | 4%  (6/167) | 14.24 | *p* = .010 | 6.19  (2.40-15.94) |
| Criminal justice system | I have a criminal record | 9%  (35/374) | 4%  (10/264) | 4.00 | *p* = 1.00 | 2.16  (1.02-4.61) |
|  | I was charged with a criminal offense (not including speeding or parking fines) | 14%  (51/374) | 8%  (22264) | 2.66 | *p* = 1.00 | 1.60  (0.91-2.81) |
|  | **I was cautioned by the police (not including cautions for minor traffic offences)** | 19%  (70/374) | 6%  (15/264) | 15.06 | *p* = .006 | 3.30  (1.90-6.02) |
|  | I spent time in prison or a juvenile detention centre | 3%  (10/374) | 2%  (5/264) | 0.02 | *p* = 1.00 | 1.09  (0.35-3.43) |
|  | I was arrested by the police | 18%  (67/374) | 9%  (23/264) | 7.03 | *p* = .482 | 2.06  (1.21-3.51) |
| Childhood victimisation | **As a child, other children bullied me** | 86%  (323/374) | 54%  (142/264) | 57.44 | *p* < .001 | 4.69  (3.15-6.10) |
|  | **As a child, an adult hurt me badly enough that it left marks on my body** | 27%  (102/374) | 17%  (44/264) | 14.02 | *p* = .010 | 2.24  (1.47-3.42) |
|  | **As a child, other children left me out of activities** | 83%  (312/374) | 47%  (123/264) | 74.46 | *p* < .001 | 5.41  (3.68-7.93) |
|  | **As a child, children spread rumours about me or talked about me behind my back** | 77%  (287/374) | 45%  (118/264) | 50.29 | *p* < .001 | 3.81  (2.63-5.52) |
|  | **As a child, another child hurt me badly enough that it left marks on my body (e·g bruises or scratches)** | 49%  (184/374) | 25%  (65/264) | 29.04 | *p* < .001 | 2.71  (1.88-3.89) |
|  | **As a child, children called me names or insulted me** | 85%  (317/374) | 57%  (150/264) | 39.89 | *p* < .001 | 3.57  (2.40-5.30) |
|  | **As a child, an adult humiliated, embarrassed or scared me** | 78%  (289/374) | 52%  (138/264) | 41.05 | *p* < .001 | 3.32  (2.30-4.78) |
|  | **As a child, an adult touched me in a sexual way, or tried to make me touch them in a sexual way** | 28%  (106/374) | 20%  (53/264) | 12.30 | *p* = .027 | 2.07  (1.38-3.11) |
|  | **As a child, an adult swore at me or called me names like stupid, ugly or lazy** | 61%  (229/374) | 33%  (87/264) | 43.43 | *p* < .001 | 3.25  (2.29-4.61) |
| Adulthood victimisation | **I have been bullied by someone in my family** | 52%  (194/374) | 34%  (89/264) | 25.26 | *p* < .001 | 2.46  (1.73-3.50) |
|  | **I have been pressured into sexual activity** | 41%  (154/374) | 22%  (59/263) | 39.99 | *p* < .001 | 3.65  (2.44-5.45) |
|  | **I have been bullied by someone at work** | 55%  (204/374) | 36%  (96/264) | 32.10 | *p* < .001 | 2.80  (1.96-4.00) |
|  | **I have been tricked or pressured into breaking the law** | 21%  (78/374) | 79%  (17/264) | 15.90 | *p* = .004 | 3.18  (1.80-5.63) |
|  | **I have been physically forced into sexual activity** | 25%  (95/374) | 15%  (39/264) | 16.19 | *p* = .003 | 2.48  (1.59-3.85) |
|  | **As an adult, I have been hurt by someone badly enough that it left marks on my body (e·g bruises or scratches)** | 33%  (123/374) | 18%  (48/264) | 18.70 | *p* < .001 | 2.43  (1.63-3.64) |
|  | **I have been bullied by someone that I considered to be a friend** | 71%  (264/374) | 31%  (83/264) | 75.00 | *p* < .001 | 5.03  (3.49-7.25) |
|  | **I have been tricked or pressured in to giving someone money or possessions** | 48%  (179/374) | 21%  (54/263) | 43.79 | *p* < .001 | 3.65  (2.49-5.35) |
| Domestic abuse | **My partner forced me into sexual activity** | 19%  (60/309) | 9%  (22/253) | 21.03 | *p* < .001 | 3.69  (2.11-6.44) |
|  | **My partner physically hurt me e·g· shoved, slapped or punched me** | 29%  (90/309) | 17%  (44/253) | 17.55 | *p* = .002 | 2.36  (1.65-3.96) |
|  | **My partner threatened to harm me or to harm someone I care about** | 22%  (67/309) | 11%  (27/253) | 14.10 | *p* = .010 | 2.64  (1.59-4.39) |
|  | **My partner took advantage of me financially** | 25%  (78/309) | 12%  (29/253) | 18.06 | *P* = .001 | 2.89  (1.77-4.71) |
|  | **My partner humiliated or embarrassed me** | 39%  (120/309) | 24%  (59/253) | 21.66 | *p* < .001 | 2.58  (1.73-3.85) |
| Mental illness | **There was a period of my life where I was regularly using alcohol or another (non-prescribed) drug in order to cope·** | 38%  (141/374) | 24%  (63/263) | 11.96 | *p* = .033 | 1.92  (1.33-2.79) |
|  | **I was incorrectly diagnosed with a mental health condition (e·g· ADHD instead of autism)** | 40%  (148/374) | 5%  (13/263) | 73.90 | *p* < .001 | 14.95  (8.07-27.69) |
|  | I have been sectioned because of a mental health condition | 8%  (31/374) | 2%  (5/263) | 9.28 | *p* = .137 | 4.63  (1.73-12.39) |
|  | **I have had a mental health condition that affected my daily life** | 82%  (306/374) | 38%  (101/264) | 99.39 | *p* < .001 | 7.48  (5.04-11.12) |
|  | **I have made suicide plans** | 57%  (214/374) | 26%  (281/263) | 53.85 | *p* < .001 | 3.91  (2.72-5.63) |
|  | **I have attempted suicide** | 40%  (148/374) | 12%  (32/263) | 46.73 | *p* < .001 | 4.66  (3.00-7.24) |
|  | **I have deliberately harmed myself** | 59%  (221/374) | 20%  (51/263) | 79.01 | *p* < .001 | 6.44  (4.27-9.71) |
| Social support | **There has always been someone in my life who would try to help me if I was in trouble** | 58%  (218/374) | 75%  (199/263) | 21.03 | *p* < .001 | 0.42  (0.29-0.61) |
|  | **There has always been someone in my life who would care for me if I was ill** | 54%  (201/374) | 74%  (194/263) | 32.87 | *p* < .001 | 0.34  (0.23-0.49) |
|  | **I have always known that there is someone in my life who loves me** | 56%  (209/374) | 76%  (200/263) | 34.53 | *p* < .001 | 0.32  (0.22-0.47) |
